# Chronic AdipoRon Treatment Mimics the Effects of Physical Exercise on Restoring Hippocampal Neuroplasticity in Diabetic Mice

**DOI:** 10.1007/s12035-021-02441-7

**Published:** 2021-06-23

**Authors:** Thomas H Lee, Ahadullah, Brian R Christie, Kangguang Lin, Parco Ming-fai Siu, Li Zhang, Ti-fei Yuan, Pragya Komal, Aimin Xu, Kwok-fai So, Suk-yu Yau

**Affiliations:** 1 Department of Rehabilitation Sciences, Faculty of Health and Social Sciences, The Hong Kong Polytechnic University, 11 Yuk Choi Road, Hung Hom, Hong Kong SAR; 2 Division of Medical Sciences, University of Victoria, Victoria, British Columbia Canada; 3 Department of Affective Disorder, Guangzhou Brain Hospital, The Affiliated Brain Hospital of Guangzhou Medical University, Guangzhou, China; 4 School of Public Health, LKS Faculty of Medicine, The University of Hong Kong, Pokfulam, Hong Kong SAR; 5 Key Laboratory of CNS Regeneration (Ministry of Education), Guangdong-Hong Kong-Macau Institute of CNS Regeneration, Jinan University, Guangzhou, China; 6 Shanghai Mental Health Center, School of Medicine, Shanghai Jiao Tong University, Shanghai, China; 7 Department of Biological Sciences, Birla Institute of Technology and Sciences (BITS-Pilani Hyderabad), Hyderabad, India; 8 Department of Pharmacology and Pharmacy, LKS Faculty of Medicine, The University of Hong Kong, Pokfulam, Hong Kong SAR; 9 State Key Laboratory of Pharmaceutical Biotechnology, The University of Hong Kong, Pokfulam, Hong Kong SAR; 10 State Key Laboratory of Brain and Cognitive Science, The University of Hong Kong, Pokfulam, Hong Kong SAR; 11 University Research Facility in Behavioral and Systems Neuroscience, The Hong Kong Polytechnic University, Hung Hom, Hong Kong SAR

**Keywords:** AdipoRon, Adiponectin, Diabetes, Adult neurogenesis, Hippocampal plasticity, Cognitive impairment, Physical exercise

## Abstract

Administration of exercise mimetic drugs could be a novel therapeutic approach to combat comorbid neurodegeneration and metabolic syndromes. Adiponectin is an adipocyte-secreted hormone. In addition to its antidiabetic effect, adiponectin mediates the antidepressant effect of physical exercise associated with adult hippocampal neurogenesis. The antidiabetic effect of the adiponectin receptor agonist AdipoRon has been demonstrated, but its potential pro-cognitive and neurotrophic effects in the hippocampus under diabetic condition are still unclear. This study reported that chronic AdipoRon treatment for 2 weeks improved hippocampal-dependent spatial recognition memory in streptozotocin-induced diabetic mice. Besides, AdipoRon treatment increased progenitor cell proliferation and neuronal differentiation in the hippocampal dentate gyrus (DG) of diabetic mice. Furthermore, AdipoRon treatment significantly increased dendritic complexity, spine density, and *N*-methyl-D-aspartate receptor-dependent long-term potentiation (LTP) in the dentate region, and increased BDNF levels in the DG of diabetic mice. AdipoRon treatment activated AMPK/PGC-1α signalling in the DG, whereas increases in cell proliferation and LTP were not observed when PGC-1α signalling was pharmacologically inhibited. In sum, chronic AdipoRon treatment partially mimics the benefits of physical exercise for learning and memory and hippocampal neuroplasticity in the diabetic brain. The results suggested that AdipoRon could be a potential physical exercise mimetic to improve hippocampal plasticity and hence rescue learning and memory impairment typically associated with diabetes.

**Supplementary Information:**

The online version contains supplementary material available at 10.1007/s12035-021-02441-7.

## Introduction

Epidemiological studies have reported that diabetes is a risk factor for developing dementia (1, 2). Rodent models of diabetes also display cognitive decline (3–6) associated with impaired adult neurogenesis in the dentate gyrus (7, 8), synaptic deficits (9, 10), and neuroinflammation (11, 12). The term type 3 diabetes signifies Alzheimer’s disease (AD) as a metabolic-cognitive syndrome, reflecting the concurrence of insulin resistance and neurodegeneration in AD brains (13). Physical exercise is an effective non-pharmacological intervention for cognitive decline in neurodegeneration and diabetes (14–16) by promoting synaptic plasticity (17) and adult hippocampal neurogenesis (18). However, chronic inactivity (19–21), mood disorder (22–24), and physical disability (25, 26) may undermine the beneficial effects of physical exercise on metabolic and brain health in the elderly under severe obese and diabetic conditions.

Activating a common molecular target that mediates the eumetabolic and pro-cognitive effects of physical exercise can be a novel therapeutic approach to combat diabetes and its associated neurodegeneration. The adipocyte-secreted hormone adiponectin is deemed to be a worthy candidate since it possesses antidiabetic property by promoting insulin sensitivity (27). Hypoadiponectinemia is restored in obese and diabetic individuals undergoing physical exercise interventions (28, 29). The central adiponectin level could be linked to neurodegeneration and cognitive decline as suggested by reduced adiponectin levels in the hippocampus of diabetic mice (30) and cerebrospinal fluid of AD patients (31). Importantly, further studies have unravelled the pivotal role of adiponectin in cognitive function and hippocampal plasticity. Learning and memory deficits are presented in adiponectin-deficient mice (32). Also, adiponectin regulates dendritic and spine formation (33) as well as synaptic plasticity (32) in the hippocampus, where adiponectin receptors 1 and 2 (AdipoR1 and AdipoR2) are expressed in the hippocampal neural progenitor cells (34), neurons (35), and synapses (32). Our team has previously shown that adiponectin mediates exercise-induced adult neurogenesis, dendritic arborisation, and AMP-activated protein kinase (AMPK) activation in the hippocampus (30, 34, 36). BDNF is a crucial downstream effector to regulate adult neurogenesis, synaptic plasticity, and cognitive function (37). The concomitant increases in brain-derived neurotrophic factor (BDNF) and adult hippocampus neurogenesis mimic the beneficial effects of voluntary running on cognitive enhancement in mice (38). Notably, activating PGC-1α signalling is required for physical exercise to increase BDNF levels (39) and dendritic spine formation (40) in the hippocampus. In addition, an early study has demonstrated that adiponectin promotes PGC-1α activity through AdipoR1/AMPK signalling (41). These studies collectively suggest that activating adiponectin signalling cascade may contribute to raising BDNF level.

Administration of AdipoRon, an agonist of AdipoR1 and AdipoR2, resembles the effects of adiponectin administration and physical exercise on improving insulin sensitivity and oxidative metabolism in diabetic mice (42). Since AdipoRon can cross the blood-brain barrier (31, 43), this study aimed to investigate whether chronic treatment with AdipoRon could act as a pro-cognitive exercise mimetic to restore hippocampal neuroplasticity in animals experiencing diabetes-associated hippocampal dysfunction.

## Materials and Methods

### Animals and Experimental Design

All experimental procedures were approved and followed the Animal Subjects Ethics Sub-Committee’s guidelines at The Hong Kong Polytechnic University. Five-week-old C57BL/6J male mice were fed with standard chow and water ad libitum and kept under a 12-h light-dark cycle. A single-dose streptozotocin (STZ; 195 mg/kg i.p.) (Santa Cruz Biotechnology Ltd., USA) was delivered intraperitoneally to induce diabetes-associated hippocampal impairment as previously described (30). Tail blood samples were measured by glucometer (Accu-Chek® Performa, Australia) 7 days after STZ administration, and only animals that exhibited hyperglycaemia (≥ 20 mmol/L) were included in the experiments (Fig. S1).

**Fig. 1:**
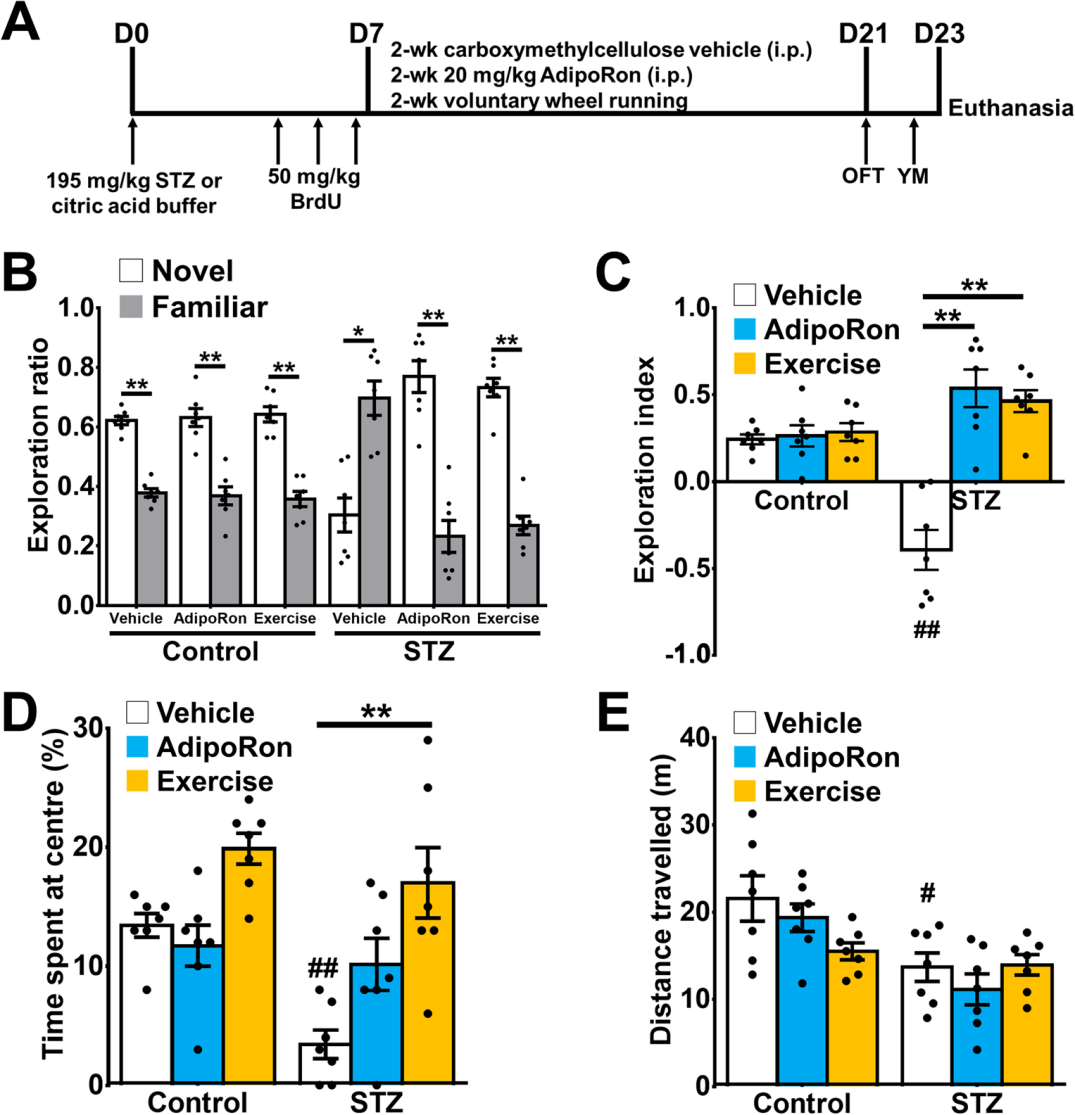
AdipoRon treatment ameliorated diabetes-associated spatial recognition memory. **A** Animal treatment timeline (*n* = 7 animals per group). Previously reported Control-Vehicle and Control-AdipoRon data were used to support this study and are available at doi: 10.3390/ijms22042068 (44). **B** Exploration ratios in Y- maze. Neither AdipoRon treatment nor exercise altered novelty preference in the Y-maze in healthy control mice. Diabetic mice showed impairment in Y-maze as shown with a greater preference towards the familiar arm (paired *t*-test: *P < 0.005 vs. Novel arm), while both AdipoRon treatment and exercise restored the memory deficit in diabetic mice (**P < 0.005 vs. Novel arm). **C** Exploration indices in Y-maze. Diabetic mice presented spatial memory deficit (Tukey’s post hoc test: ^##^*P* < 0.005 vs. Control-Vehicle), whereas AdipoRon treatment (Tukey’s post hoc test: **P < 0.005 vs. STZ-Vehicle) and exercise (Tukey’s post hoc test: **P < 0.005 vs. STZ-Vehicle) ameliorated memory deficit. **D** Diabetic mice showed anxiety-like behaviour (Tukey’s post hoc test: ^##^*P* < 0.005 vs. Control-Vehicle), which could be restored by exercise (Tukey’s post hoc test: **P < 0.005 vs. STZ-Vehicle). **E** Diabetic mice had reduced locomotor activity (Tukey’s post hoc test: ^#^*P* < 0.05 vs. Control-Vehicle). AdipoRon treatment and exercise (Tukey’s post hoc test: *P* > 0.05 vs. STZ-Vehicle) did not improve locomotor activity in diabetic mice

AdipoRon (20 mg/kg in 0.5% carboxymethylcellulose salt solution, i.p. injection) (Sigma-Aldrich, MO, USA) or vehicle was delivered continuously for 14 days (44). In the voluntary exercise regime, mice were group housed in the holding cage, with shared running wheels as previously described (30, 34). Mice were injected with bromodeoxyuridine i.p. (50 mg/kg in 0.9% saline) before AdipoRon treatment to label surviving new-born cell in the DG. The day after the last treatment, mice were subjected to the open field test and Y-maze task (Fig. 1A).

### Behavioural Tests

#### Open Field Test

Mice were brought to the testing room 2 h prior to the test for adaptation (44). Each mouse was allowed to explore the open field (L × W × H: 40 × 40 × 30 cm) for 10 min in dim light. Anxiety-like behaviour and locomotor activity were analysed by ANYMAZE software (Stoelting Co., IL, USA). Locomotor activity was presented as the total distance travelled over 10 min.

#### Y-Maze Task

Three identical arms (L × W × H: 10 × 6 × 8 cm) in Y-maze were pre-designated as the starting arm, the familiar arm, and the novel arm, respectively (44). Three arms were counterbalanced to avoid recognition bias. Tests were carried out in the dim light setting. In the 10-min habituation session, a mouse was released in the designated starting arm and was allowed to explore the maze with a blocked novel arm. After a 4-h intermission, the mouse was allowed to explore the maze with the unblocked novel arm for 5 min. Time spent in each arm was hand-scored by the trained researcher in a sample-blinded manner. Exploration ratios were calculated as (time spent in the novel arm (N)/(time spent in novel + familiar arms) and (time spent in the familiar arm (F))/(time spent in novel + familiar arms), and an exploration index (in a −1 to 1 scale) was calculated as (N−F)/(N+F).

### Tissue Preparation for Immunostaining

Mice were deeply anaesthetised with isoflurane (Zoetis, UK). Blood samples were collected transcardially by using a 1-mL syringe with a 25-G needle. Blood samples were clotted for 30 min at room temperature, and blood serum samples were collected by centrifugation at 1,000*g* for 20 min at 4 °C, and then stored at −80 °C until measurements using enzyme-linked immunosorbent assay (ELISA). Animals were then perfused with 0.9% saline, followed by 4% paraformaldehyde (PFA) in 0.01 M phosphate-buffered saline (PBS). The isolated brains were post-fixed with 4% PFA at 4 °C overnight. The brains were then stored in 30% sucrose until they sank. Coronal sections (1-in-6 series, 30-μm thickness) were prepared using a vibratome (Leica Biosystems, Germany). The brain slices were stored in cryoprotectant (30% glycerol and 30% ethylene glycol) at 4 °C until immunostaining.

#### Immunohistochemistry

Immunostaining was performed by the free-floating method as previously performed (44). For BrdU staining, the antigens were retrieved in the citric acid buffer (pH 6.0) at 95 °C for 15 min, then denatured in 2 N HCl for 30 min and neutralised by 0.1 M borate buffer (pH 8.5) for 15 min at room temperature. The sections were incubated overnight with mouse anti-BrdU antibody (1:1,000; Roche Life Science, Germany) and then incubated with the biotinylated goat anti-mouse IgG (1:200; Vector Laboratories, CA, USA) for 2 h at room temperature. For doublecortin (DCX) and Ki-67 staining, sections were incubated with mouse anti-DCX (1:200; Santa Cruz Biotechnology, TX, USA) or rabbit anti-Ki67 (1:1,000; Abcam, UK) antibodies, then secondary antibodies: biotinylated goat anti-mouse or goat anti-rabbit IgG (1:200; Vector Laboratories, CA, USA). Positive staining was visualised with the peroxidase method using the VECTASTAIN® ABC kit (HRP) (Vector Laboratories, CA, USA) and the DAB peroxidase substrate kit (Vector Laboratories, CA, USA).

#### Immunofluorescent Co-labelling

Immunofluorescent co-labelling of BrdU and DCX was performed as previously described (34, 44). After antigen retrieval, sections were incubated with primary antibodies overnight and secondary antibodies: goat anti-rabbit IgG Alexa Fluor-488 and goat anti-mouse IgG Alexa Fluor-568 for 2 h at room temperature. After washes with PBS, the sections were counterstained by DAPI and coverslipped with the fluorescent mounting medium (Dako, CA, USA).

#### Quantifications of BrdU+, Ki67+, and DCX+ cells

Cells were counted in the 1-in-6 series of nine sections (from bregma −1.34 to −3.80 mm), using optical fractionators system (grid size: 55 μm × 55 μm; counting frame: 35 μm × 35 μm) of Stereo Investigator (MicroBrightField Inc., VT, USA) as previously described (44). Cells located in the dentate subgranular zone and granular cell layer were counted, whereas those located in the uppermost focal plane were excluded. Quantification was performed in a sample-blinded manner.

#### Quantifications of DCX+/BrdU+ co-labelled cells

Images of six sections from each animal were captured using LSM 800 confocal laser scanning microscope (Carl Zeiss Microscopy, NY, USA). Fifty BrdU positive cells were randomly selected, followed by confirming the DCX co-labelled ratio to indicate neuronal differentiation (34, 45). Quantification of the co-labelling was performed in a sample-blinded manner (30, 44).

### Golgi Staining

Golgi staining was performed using the commercially available FD Rapid GolgiStain™ Kit (FD Neurotechnologies, Inc., MD, USA). Solutions A and B in FD Rapid GolgiStain™ Kit were pre-mixed in a 1:1 ratio for a week. Mice were deeply anaesthetised by isoflurane (Zoetis, UK) and rapidly decapitated to obtain the whole brain. The hemispheres were halved and kept in solution A–B mixture in the dark at room temperature for 6 days. Then, the halved brains were immersed in cryoprotectant at 4 °C in the dark until they sank. Cryoprotectant was replaced after the first 24 h. Coronal brain slices with 200-μm thickness were obtained from rostral to caudal (from bregma −1.34 to −3.80 mm) using a vibratome (Leica Biosystems, Germany) filled with ice-cold, 6% sucrose solution. Sections were immediately mounted onto a chrome alum-coated slide with solution C and dried in a dark chamber overnight at room temperature. The staining solution was freshly prepared by mixing solution D, solution E, and Milli-Q water in 1:1:2 ratio. Samples were stained for 10 min and were briefly rinsed by Milli-Q water. At last, dried samples were dehydrated through 70%, 90%, and 100% ethanol (5 min each, 10 min for absolute ethanol) and xylene for 15 min. Samples were then coverslipped. 

#### Dendritic Branch Analyses

Granule cells were selected based on the following criteria (45–48): (1) Neuronal cell bodies located in the middle part of the section thickness to minimise the truncated branch segments; (2) consistent impregnation along with the entire extent of all dendrites; (3) located in the appropriate subregion of the dorsal hippocampi. Specifically, granule cells in the hippocampal DG were selected based on their soma position because granule cells with different soma position in the dentate gyrus could have different morphologies. Granule cells located closer to the subgranular zone show a simpler dendritic architecture with typically one primary dendrite extension. The granule cells located in the outer granule cell layer display multiple primary dendritic extensions and have more complex dendritic branching. Single-branched granule cells were selected from the inner cell layer, and multiple-branched granule cells were selected from the outer cell layer as previously performed (45–48). Five single-branched and five multiple-branched granule cells were selected from each sample to measure the total dendritic length, and Sholl analysis was performed using Neurolucida (MicroBrightField Bioscience, VT, USA). 

#### Dendritic Spine Analyses

Five single-branched and five multiple-branched granule cells were selected from each sample. Five segments of tertiary or quaternary dendritic branches (longer than 15 μm) were selected from a granule cell using ×400 magnification under a light microscope (Axioplan, Zeiss, Oberkochen, Germany) (45, 47). Quantification of dendritic spines was performed using ×630 magnification. 

### Protein Extraction

Mice were rapidly decapacitated. The hippocampal DG was isolated from the Ammon’s horn as previously described (49). The dentate gyrus samples were homogenised from a pool of seven mice from the same treatment group in ice-cold radioimmunoprecipitation assay buffer (Abcam, Cambridge, UK) containing Halt© phosphatase/protease cocktail (Thermo Fisher Scientific, Waltham, MA, USA) and phenylmethanesulfonyl fluoride (Thermo Fisher Scientific, Waltham, MA, USA). Samples were then sonicated for 20 s with a 50% pulse. Supernatants were collected by centrifugation (14,000*g*) at 4 °C for 30 min. Protein concentrations of supernatant were quantified by Bradford assay (Bio-Rad Laboratories, Hercules, CA, USA) and stored at −80 °C.

#### Western Blotting

Homogenates were heat linearised in 1× laemmli buffer (Bio-Rad Laboratories, Hercules, CA, USA) with 5% β-mercaptoethanol (Abcam, Cambridge, UK) for 10 min. Homogenate containing 50 μg protein was loaded in a lane. Proteins were separated by 10% TGX stain-free FastCast© SDS gel (Bio-Rad Laboratories, Hercules, CA, USA) and transferred to polyvinylidene fluoride (PVDF) membranes (Bio-Rad Laboratories, Hercules, CA, USA). Blocking solution contains 5% non-fat dry milk powder (Bio-Rad Laboratories, Hercules, CA, USA) for non-phosphorylated protein targets or 5% bovine serum albumin (Sigma, MO, USA) for phosphor-protein targets in the Tris-HCl buffer (pH 8.0). After 1-h blocking, membranes were incubated overnight with primary antibodies in blocking solution with 0.05% Tween 20. Primary antibodies from the rabbit are used: pAMPKα^Thr172^ (1:1,000, Cell Signaling), AMPKα (1:1,000, Cell Signaling), pPGC1α^Ser571^ (1:1,000, R&D systems), PGC1-α (1:1000, Millipore), β-actin (1:5,000, Cell Signaling). After three washes, membranes were probed for 1 h with the horseradish peroxidase-conjugated secondary antibodies. After the final three washes, bands were developed by enhanced chemiluminescent (ECL) detection kit (Santa Cruz Biotechnology, Inc., Dalla, TX, USA) and documented by a transilluminator (Bio-Rad Laboratories, Hercules, CA, USA). Band intensities were subjected to densitometric analysis in the Image Lab software (Bio-Rad Laboratories, Hercules, CA, USA).

### Tissue Preparation for Electrophysiology

Brain slices were prepared as previously described (50). Adult male animals (8–9 weeks old) were deeply anaesthetised with isoflurane and rapidly decapitated. The brains were isolated and immersed in a chilled artificial cerebrospinal fluid (ACSF) containing (in mM) 125 NaCl, 25 NaHCO_3_, 3 KCl, 1.25 NaH_2_PO_4_, 10 D-glucose, 1 CaCl_2_, and 6 MgCl_2_, and saturated with 95% O_2_/5% CO_2_. Transverse brain slices (thickness: 350 μm) were obtained using a semi-automatic vibratome (VT1000S, Leica Biosystems Inc., IL, USA). Slices were gently transferred to an incubation chamber filled with ACSF containing (in mM) 125 NaCl, 25 NaHCO_3_, 3 KCl, 1.25 NaH_2_PO_4_, 10 D-glucose, 2 CaCl_2_, and 1 MgCl_2_, and saturated with 95% O_2_/5% CO_2_. Slices were allowed to recover at 35 °C for a minimum of 1 h before recording. 

#### Long-term Potentiation Field Recording

Hippocampal DG was positioned on the 64 extracellular electrodes in a recording probe (P515A, Alpha MED Scientific Inc., Osaka, Japan) to stimulate granule neurons in the middle molecular layer (medial perforant pathway) of the suprapyramidal blade. Recordings of field excitatory postsynaptic potentials (fEPSPs) were made on adjacent channels located midway between the crest and the distal end of the limb. Slices were perfused at a rate of approximately 2 mL/min. fEPSPs were acquired using the recording amplifiers (MED-A64MD1 and MED-A64HE1S, Alpha MED Scientific Inc., Osaka, Japan) connected to a Windows computer running Mobius software. For each slice, stimulus intensity (20–35 μA) was adjusted to yield 40–50% of the maximal response slope without population spikes. Baselines fEPSP measurements were obtained by delivering single-pulse stimulation at 15-s interstimulus intervals. After a steady baseline of at least 20 min, a conditioning protocol was used to induce synaptic plasticity, followed by baseline measurement for 1 h as previously performed (50). High-frequency stimulation (HFS) protocol (4 trains of 50 pulses, delivered at 100 Hz with 30-s intertrain interval) was applied in the presence of bicuculline methiodide (5 μM) to inhibit the γ-aminobutyric acid-A (GABA_A_) receptors and facilitate LTP induction in the DG. In pharmacological intervention, brain slices were acutely perfused in PEAQX tetrasodium hydrate (0.1 μM; NVP-AAM077) (Sigma-Aldrich, MO, USA) to inhibit GluN2A subunit (51) or in Ro 25-6981 maleate (0.5 μM) (Alomone Labs, Israel) to inhibit GluN2B subunit (52) for 30 min before HFS. Similarly, SR-18292 maleate (1 nM; Sigma-Aldrich, MO, USA), a PGC-1α blocker, was perfused 20 min before HFS. AdipoRon (500 nM dissolved in aACSF) was bath perfused in the last 10 min in the presence of inhibitors. 

### Immunoassays for Adiponectin and BDNF Levels

Levels of adiponectin and BDNF in the collected sera and dentate gyri were determined by using the commercial ELISA kits, including mouse adiponectin ELISA kit for measuring serum adiponectin levels (Immunodiagnostics, University of Hong Kong, Hong Kong), mouse adiponectin ELISA kit for measuring adiponectin levels in the hippocampal DG (AdipoGen Life Sciences, Switzerland), and the total BDNF Quantikine ELISA kit (R&D system, MN, USA) according to the manufacturer’s instructions.

### N2a Mouse Neuroblastoma Cell Line Maintenance

Murine neuroblastoma cell line N2a (ATCC, VA, USA) was maintained in the Gibco® Dulbecco’s modified Eagle’s medium (DMEM, Thermo Fisher Scientific, MA, USA) containing 10% foetal bovine serum (FBS), 2 mM L-glutamine, and 50 μg/mL penicillin-streptomycin. After reaching 90% confluence, cell cultures were washed with Gibco® Dulbecco’s PBS (DPBS, Thermo Fisher Scientific, MA, USA) and incubated with the mixture of 0.25% trypsin/0.02% EDTA at 37 °C for 10 min. Trypsinization was halted by FBS-containing DMEM. The cell suspension was pelleted at 300*g* for 7 min and split in four. Passages 3–7 (the initial cell stock as passage 0) were used for the following assays.

### Cell Proliferation Assay

CyQUANT^TM^ NF Cell Proliferation Assay Kit (Invitrogen, MA, USA) was used to examine the proliferative effect of AdipoRon in vitro*.* CyQuant® dye is a fluorochrome that intercalates with the nuclear DNA content in living cells (53). The emission intensity is correlated linearly with cell number over the range of 10 to 50,000 cells (53). One hundred microliters trypsinised subculture was seeded at a density of 0.02 million per well in the 96-well flat-bottom microplates (Falcon, Fisher Scientific, NH, USA) coated with 0.1% poly-L-lysine (Sigma, MO, USA). The subculture was maintained in the drug-free culture medium for 24 h. The next day, the medium was decanted and replaced by a new medium containing AdipoRon alone or with the inhibitors. Specifically, AdipoRon (40 mg/mL), GW9662 (20 mg/mL), and SR-18292 maleate (20 mg/mL) were dissolved in DMSO as stocks and then diluted with the culture medium. To examine the proliferative effect of AdipoRon, subculture was independently treated by AdipoRon (0.5, 1, 5, or 25 μM) for 4 h or 24 h. To examine the involvement of PPAR-γ and PGC-1α in AdipoRon-induced cell proliferation, subculture was first pre-treated by inhibitors PPAR-γ blocker: GW9662 (10 μM; APE×BIO, TX, USA) (54) and PGC-1α blocker: SR-18292 (20 μM; Sigma-Aldrich, MO, USA) (55) for 1 h, followed by AdipoRon and inhibitor co-treatment for 24 h. All control samples were treated with 2% DMSO (v/v) as vehicle treatment. After treatments, culture media were decanted. One hundred microliters 1× CyQuant® NF dye was added to each well. One hour after, sample fluorescence (excitation/emission λ = 480/520 nm) was measured using the Varioskan^TM^ Flash Plate Reader (Thermo Fisher Scientific, MA, USA). Cell proliferation rate was determined by comparing emission intensity for a drug-treated subculture to DMSO-treated controls. The relative cell proliferation was calculated as follows:$\mathrm{Cell}\ \mathrm{proliferation}\%=\frac{\left({\mathrm{OD}}_{\mathrm{Sample}}-{\mathrm{OD}}_{\mathrm{Blank}}\right)}{\left({\mathrm{OD}}_{\mathrm{Control}}-{\mathrm{OD}}_{\mathrm{Blank}}\right)}\times 100\%$, where readings of the cell-free well and untreated wells were the blank and the positive control, respectively. 

### Statistical Analyses

Data were shown as mean ± SEM. A paired *t*-test was performed to compare the novel/familiar arm visit in the Y-maze task. To evaluate the effects of AdipoRon treatment and STZ-diabetes, two-way ANOVA was employed in behavioural assessments, histological analyses, fEPSP analyses, and ELISA by Tukey post hoc test. Wilcoxon matched-pairs signed-rank test between AdipoRon- and vehicle-treated animals in the control and diabetic animals separately to address dendritic complexity. Two-way ANOVA with LSD post hoc test was applied in the densitometric analyses of immunoblotting. One-way ANOVA with LSD post hoc test was used to evaluate the AdipoRon dosage effect in in vitro proliferation assay. Statistical analyses were performed in Prism 8.0 software (GraphPad Software, USA). *P* < 0.05 was considered statistically significant.

## Results

### Chronic Treatment with AdipoRon Mimicked Voluntary Running to Ameliorate Diabetes-Impaired Spatial Memory

Hippocampal-dependent spatial recognition memory was assessed by a Y-maze test (56). Control mice with an intact spatial recognition memory showed a significantly higher exploration ratio in the novel arm (Fig. 1B and Appendix Table S1; *P* < 0.0005 vs. Novel arm). The STZ-diabetic mice showed a greater preference towards the familiar arm (Fig. 1B; *P* = 0.0140 vs. Novel arm), suggesting spatial memory deficit in diabetic mice. Two-way ANOVA revealed significant main effects of treatments (effect of interaction: *F*_*2,36*_ = 21.01, *P* < 0.0001; effect of STZ: *F*_*1,36*_ = 0.97, *P* = 0.331; effect of treatment: *F*_*2,36*_ = 23.99, *P* < 0.0001). AdipoRon treatment (Fig. 1C and Appendix Table S2; *P* < 0.0001 vs. STZ-Vehicle) mimicked the effect of voluntary running (Fig. 1C; *P* < 0.0001 vs. STZ-Vehicle) on restoring spatial memory deficits.

Diabetic mice had a shorter time spent at the centre in the open field (Fig. 1D; *P* < 0.005 vs. Control-Vehicle), indicating an increased in anxiety-like behaviour (effect of interaction: *F*_*2,36*_ = 2.998, *P* = 0.0625; effect of STZ: *F*_*1,36*_ = 10.09, *P* = 0.0031; effect of treatment: *F*_*2,36*_ = 15.75, *P* < 0.0001). Voluntary running elicited anxiolytic effect on diabetic animals since diabetic runners spent significantly more time in the centre of the open field (Fig. 1D; *P* = 0.0001 vs. STZ-Vehicle). The anxiolytic effect was not observed in AdipoRon-treated diabetic mice (Fig. 1D; *P* = 0.133 vs. STZ-Vehicle). Neither running (Fig. 1E; *P* > 0.99 vs. STZ-Vehicle) nor AdipoRon treatment (Fig. 1E; *P* = 0.895 vs. STZ-Vehicle) affected locomotor activity.

### AdipoRon Treatment Resembles Voluntary Running on Restoring Hippocampal Adult Neurogenesis in Diabetic Mice

**Fig. 2:**
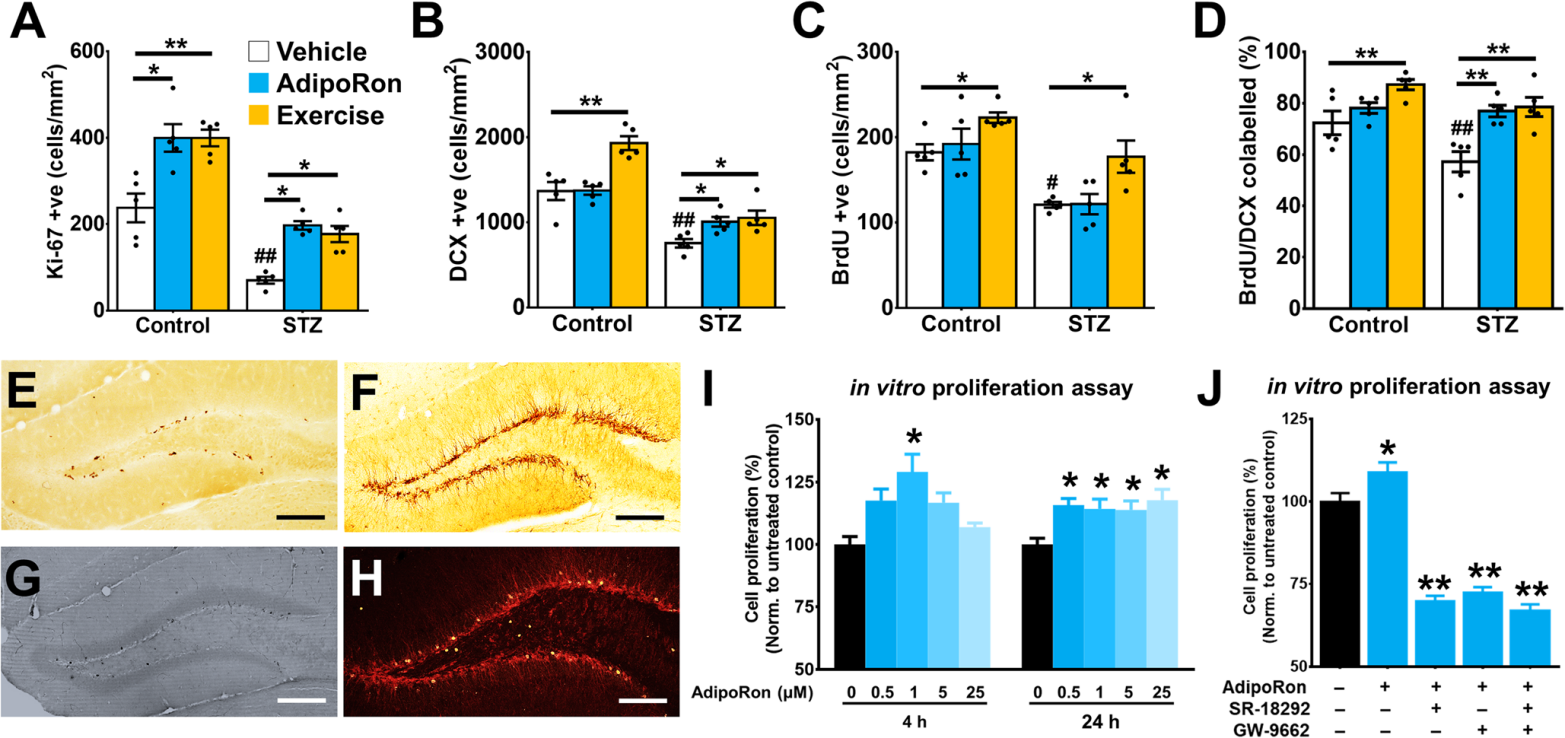
AdipoRon treatment mimicked the effect of physical exercise in restoring adult hippocampal neurogenesis in diabetic mice. **A** STZ-diabetes reduced number of Ki-67^+^ proliferating cell (Tukey’s post hoc test: ^##^*P* < 0.005 vs. Control-Vehicle). AdipoRon treatment (Tukey’s post hoc test: *P < 0.05 vs. Control-Vehicle; *P < 0.05 vs. STZ-Vehicle) mimicked the effect of exercise (Tukey’s post hoc test: **P < 0.005 vs. Control-Vehicle; *P < 0.05 vs. STZ-Vehicle) on increasing proliferating cell in both control and diabetic mice. *n* = 5 per group. Previously reported Control-Vehicle and Control-AdipoRon data were used to support this study (44). **B** STZ-diabetes reduced the density of DCX^+^ immature neuron (Tukey’s post hoc test: ^##^*P* < 0.005 vs. Control-Vehicle). AdipoRon treatment (Tukey’s post hoc test: **P < 0.005 vs. STZ-Vehicle) restored the density of immature neurons as running did in diabetic mice (Tukey’s post hoc test: **P < 0.005 vs. STZ-Vehicle), but not increasing the immature neuron density in control mice (Tukey’s post hoc test: *P > 0.05 vs. Control-Vehicle). Previously reported Control-Vehicle and Control-AdipoRon data were used to support this study (44). **C** STZ reduced cell survival in the DG (Tukey’s post hoc test: ^#^*P* < 0.05 vs. Control-Vehicle) marked by BrdU. Voluntary running increased cell survival in both control (Tukey’s post hoc test: *P < 0.05 vs. Control-Vehicle) and diabetic animals (Tukey’s post hoc test: *P < 0.05 vs. STZ-Vehicle). However, AdipoRon treatment showed no significant effects (Tukey’s post hoc test: *P > 0.05 vs. STZ-Vehicle). Previously reported Control-Vehicle and Control-AdipoRon data were used to support this study (44). **D** STZ impaired neuronal differentiation (Tukey’s post hoc test: ^#^*P* < 0.05 vs. Control-Vehicle) with reduced percentages of BrdU/DCX co-labelling. AdipoRon treatment (Tukey’s post hoc test: **P < 0.005 vs. STZ-Vehicle) restored neuronal differentiation of new-born cells in diabetic mice (Tukey’s post hoc test: ^#^*P* < 0.05 vs. Control-Vehicle), as runners did. However, AdipoRon did not show effects on neuronal differentiation in healthy control mice (*n* = 5 animals per group). Previously reported Control-Vehicle and Control-AdipoRon data were used to support this study (44). **E**–**H** Representative image of **E** proliferating progenitor cell, marked by Ki-67, in the subgranular zone. **F** Representative image of immature neurons, marked by doublecortin (DCX). **G** New-born cell (3 days before treatment until day of sacrifice), marked by BrdU, in DG. **H** Representative image of BrdU/DCX co-labelled cells, representing the new-born cells committing the neuronal lineage. **I** In vitro study of the direct effect of AdipoRon on cell proliferation in culture N2a cells. AdipoRon exerted proliferating effect in a time- and dose-dependent manner (6 replicates per independent experiment; *n* = 3 independent experiments/treatment group) in CyQuant assay (LSD post hoc test: *P < 0.05 vs. Control). **J** In vitro examination of the involvement of PPAR-γ/PGC-1α signalling in AdipoRon-induced cell proliferation in culture N2a cells. AdipoRon-induced cell proliferation was mediated by PPAR-γ/PGC-1α signalling. Blocking either PPAR-γ by SR-18292 or PGC-1α by GW9662 or co-treatment diminished AdipoRon-induced cell proliferation (9 replicates per independent experiment; *n* = 4 independent experiments/group) (LSD post hoc test: *P < 0.05 vs. untreated control)

We have previously shown that voluntary running restored adult neurogenesis in STZ-diabetic mice (30). Here we sought to determine whether AdipoRon treatment could mimic the effect of voluntary running on promoting adult hippocampal neurogenesis. AdipoRon treatment showed similar effect to voluntary running on promoting cell proliferation (Fig. 2A and E; *P* = 0.0004 vs. Control-Vehicle), but not survival (Fig. 2C and G; *P* = 0.99 vs. Control-Vehicle) or differentiation (Fig. 2D and H; *P* = 0.99 vs. Control-Vehicle) of new-born neurons in the control mice. Voluntary wheel running significantly increased the density of immature neuron (Fig. 2B and F; *P* = 0.0002 vs. Control-Vehicle) and neuronal differentiation (Fig. 2D; *P* = 0.0414 vs. Control-Vehicle) in control mice. In diabetic mice, AdipoRon treatment mimicked the effect of voluntary running on restoring proliferating cells (Figs. 2A and S2A; *P* = 0.0063 vs. STZ-Vehicle), immature neurons (Figs. 2B and S2B; *P* < 0.0005 vs. STZ-Vehicle), and promoting neuronal differentiation (Figs. 2D and S2D; *P* = 0.0034 vs. STZ-Vehicle), but not cell survival (Figs. 2C and S2C; *P* = 0.99 vs. STZ-Vehicle).

### Involvement of PPAR-γ/PGC-1α Signalling in the Proliferative Effect of AdipoRon In Vitro

Adiponectin significantly enhances cell proliferation in cultured N2a cells (34). We, therefore, examined whether AdipoRon treatment exerts a direct effect on promoting cell proliferation using N2a cell culture. Here we showed that an acute and dose-specific effect of AdipoRon (1 μM, 4-h incubation) on promoting cell proliferation (Fig. 2I; *P* < 0.05 vs. 0 μM AdipoRon). However, prolonged incubation (24 h) at all AdipoRon dosages ranging from 0.5 to 25 μM promoted cell proliferation (Fig. 2I; *P* < 0.05 vs. 24 h: 0 μM AdipoRon), suggesting a time-dependent effect of AdipoRon on promoting cell proliferation.

The AMPK/PPAR-γ/PGC-1α signalling pathway is involved in the anti-obesity effects of adiponectin (57, 58), while both adiponectin (34, 59–61) and physical exercise (34, 36) activate the AMPK signalling pathway. Therefore, we further examined whether AdipoRon treatment promotes cell proliferation via activating AMPK/PPAR-γ/PGC-1α signalling in vitro. The results indicated that treatment with PGC-1α inhibitor SR-18292 or PPAR-γ inhibitor GW9662 alone, or together, significantly inhibited the proliferative effect of AdipoRon on cell proliferation (Fig. 2J; *P* < 0.005 vs. Control). The data suggest that PPAR-γ/PGC-1α signalling is critical for AdipoRon to promote cell proliferation.

### AdipoRon Treatment Ameliorated Impairments in Dendritic Arborisation and Spine Loss in Diabetic Mice

**Fig. 3:**
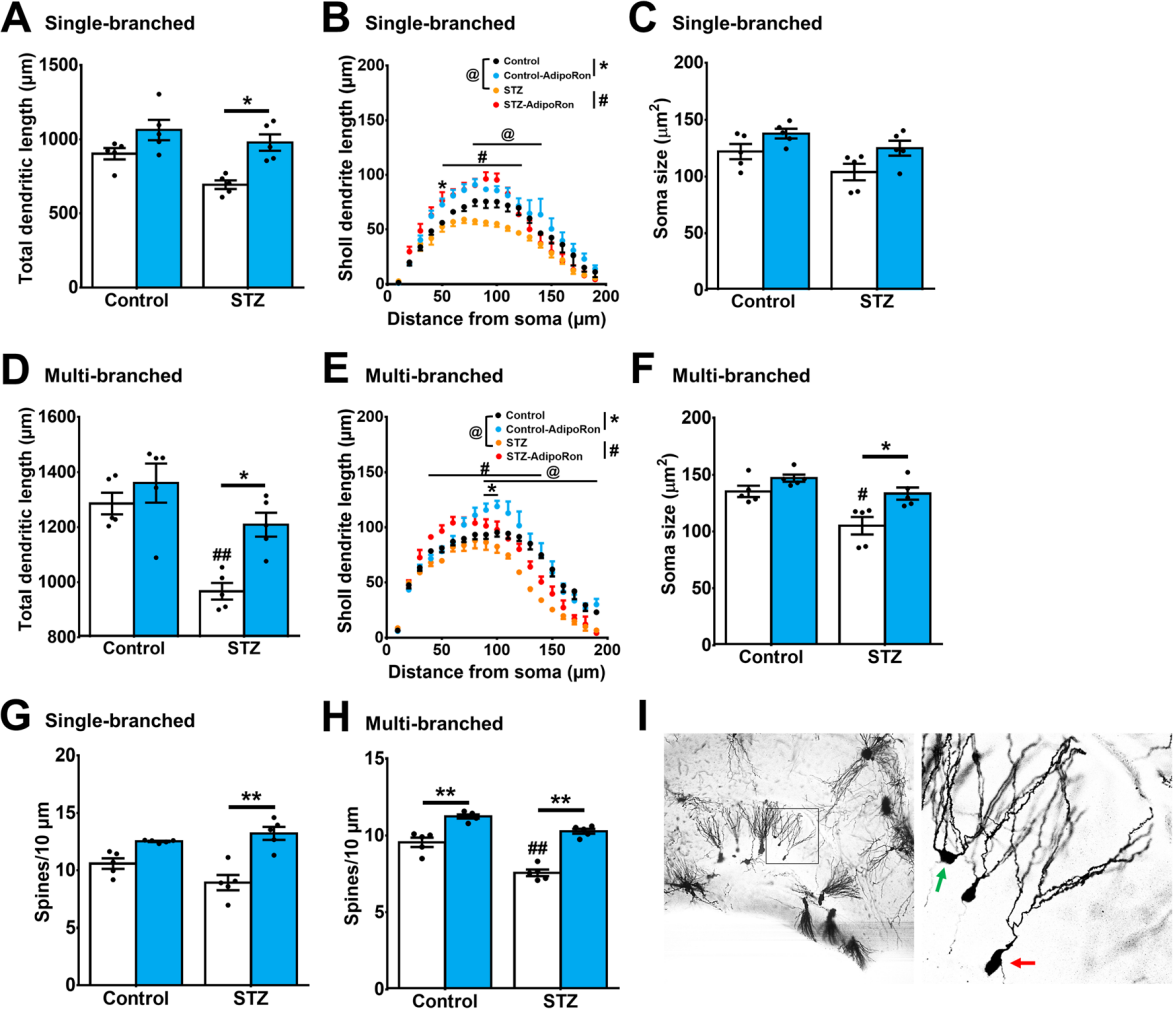
AdipoRon restored dendritic arborisation, soma size, and spine density of dentate granule cells in diabetic mice. **A**, **B** In granule cells with single primary dendrite, STZ reduced **A** total dendritic length (Tukey’s post hoc test: ^#^*P* < 0.05 vs. Control-Vehicle) and **B** dendritic arborisation in diabetic mice (Tukey’s post hoc test: ^#^*P* < 0.05 vs. STZ-Vehicle), which could be restored by AdipoRon treatment (Tukey’s post hoc test: *P < 0.05 vs. STZ-Vehicle). **C** Diabetic condition and AdipoRon treatment did not alter soma size in single-branched neurons. **D**, **E**: **D** In granule cells with multiple primary dendrites, STZ reduced total dendritic length (Tukey’s post hoc test: ^##^*P* < 0.005 vs. Control-Vehicle). Meanwhile, AdipoRon treatment restored total dendritic length (Tukey’s post hoc test: *P < 0.05 vs. STZ-Vehicle) and **E** promoted dendritic arborisation in diabetic mice (Tukey’s post hoc test: ^#^*P* < 0.05 vs. STZ-Vehicle), whereas AdipoRon restored the dendritic length (Tukey’s post hoc test: *P < 0.05 vs. STZ-Vehicle). **F** AdipoRon treatment restored reduction in soma size in diabetic mice (Tukey’s post hoc test: ^#^*P* < 0.05 vs. Control-Vehicle, *P < 0.05), but AdipoRon did not affect soma size in multiple-branched neurons of control mice. **G** AdipoRon treatment increased spine density in the granule cells with single primary dendrite in diabetic mice (Tukey’s post hoc test: **P < 0.005 vs. STZ-Vehicle). **H** Diabetes reduced spine density in granule neurons with multiple primary dendrites (Tukey’s post hoc test: ^##^*P* < 0.005 vs. Control-Vehicle), which could be restored by AdipoRon treatment in both control and diabetic mice (Tukey’s post hoc test: **P < 0.005). *n* = 5 animals per group. **I** Representative Golgi staining of the hippocampal dentate gyrus, with green arrow marking the multiple-branched (more mature) neuron at the outer granule cell layer, and the red arrow marking a single-branched (late-born) neuron at the inner granule cell layer

Structural changes in dendritic arborization could also affect hippocampal function; and hence hippocampal-dependent learning and memory (45, 62, 63). Voluntary running exercise significantly promotes dendritic enrichment in the hippocampus (18, 45, 64). The Golgi-stained neurons in diabetic mice showed significant reductions in total dendritic length in both younger (Fig. 3A; *P* = 0.0434 vs. Control-Vehicle) and mature granule neurons (Fig. 3D; *P* = 0.0014 vs. Control-Vehicle). Sholl analysis revealed that AdipoRon treatment enhanced dendritic complexity in diabetic mice (Figs. 3B and S3A; *P* = 0.0012 vs. STZ-Vehicle and Figs. 3E and S3B; *P* = 0.0003 vs. STZ-Vehicle). Neither STZ (Fig. 3C; *P* = 0.2215 vs. Control-Vehicle) nor AdipoRon (Fig. 3C; *P* = 0.323 vs. Control-Vehicle) affected soma size in younger granule neurons. Two-way ANOVA revealed a significant main effect of diabetes on soma size in mature granule cells (Fig. 3F; effect of interaction: *F*_*1,16*_ = 2.251, *P* = 0.153; effect of STZ-diabetes: *F*_*1,16*_ = 15.64, *P* = 0.0011; effect of treatment: *F*_*1,16*_ = 12.97, *P* = 0.0024). AdipoRon significantly restored soma size in mature granule cells in diabetic mice (Fig. 3F; *P* = 0.0114 vs. STZ-Vehicle). Similarly, two-way ANOVA revealed significant main effects of AdipoRon and diabetes on spine density in mature granule cells (Fig. 3G; effect of interaction: *F*_*1,16*_ = 5.826, *P* = 0.0281; effect of STZ: *F*_*1,16*_ = 48.04, *P* < 0.0001; effect of treatment: *F*_*1,16*_ = 106.3, *P* < 0.0001). Diabetes reduced spine density in mature granule cells (Figs. 3H and S4B; *P* < 0.0001 vs. Control-Vehicle) but not the younger neurons (Figs. 3G and S4A; *P* = 0.124 vs. Control-Vehicle). Notably, AdipoRon treatment increased spine density in mature granule neurons of both control and diabetic mice (Fig. 3H–I; Control-AdipoRon: *P* = 0.0002 vs. Control-Vehicle and STZ-AdipoRon: *P* < 0.0001 vs. STZ-Vehicle).

### AdipoRon-Restored LTP Deficits in the Hippocampal DG of Diabetic Mice

**Fig. 4:**
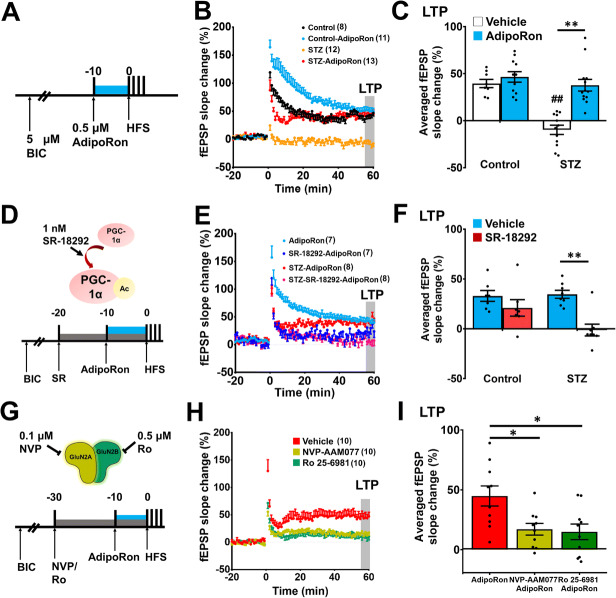
AdipoRon treatment restored NMDA receptor-dependent LTP deficit in diabetic mice depending on PGC-1α signalling. **A** LTP induction using HFS protocol to examine the effect of AdipoRon on synaptic plasticity in the hippocampal dentate gyrus. **B** Effect of AdipoRon on field excitatory postsynaptic potential (fEPSP) in control and diabetic brain slices. **C** The averaged fEPSP slope change of the last 5 min. Brain slice from diabetic mice showed deficit in LTP formation, which was rescued by 0.5 μM AdipoRon (Control: *n* = 8 slices; Control-AdipoRon: *n* = 11 slices; STZ: *n* = 12 slices; STZ-AdipoRon: *n* = 13 slices; Tukey’s post hoc test: *P < 0.005 Vehicle vs. AdipoRon; **P < 0.005 between Vehicle vs. AdipoRon; ##*P* < 0.005 Control vs. STZ). **D** Experimental protocol of PGC-1α inhibition. **E**, **F**: **E** Effect of PGC-1α inhibition on AdipoRon-elicited field excitatory postsynaptic potential. **F** The average fEPSP slope change of the last 5 min where inhibition of PGC-1α significantly impaired LTP formation (Tukey’s post hoc test: **P < 0.005 between Vehicle vs. SR-18292 under STZ condition). *n* = 7 slice per control group and *n* = 8 slices per STZ-diabetic group. **G** Experimental protocol for investigating the involvements of NMDA receptor subunits (GluN2A and GluN2B) in AdipoRon-induced LTP in diabetic slices. **H**, **I**: **H** Inhibition of GluN2A or GluN2B diminished AdipoRon-restored field excitatory postsynaptic potential in diabetic brain slices. **I** The average fEPSP slope change of the last 5 min where inhibition of GluN2A or GluN2B diminished LTP formation (*n* = 10 slices per treatment group; Tukey’s post hoc test: *P < 0.05 NVP-AAM077 or Ro-25-6981 vs. Vehicle)

Previous studies have shown that long-term voluntary wheel running exercise (2 to 3 months) promotes hippocampal synaptic plasticity (62, 65). To determine whether AdipoRon promotes synaptic plasticity as exercise does, we examined its effect on LTP formation in the hippocampal DG of diabetic mice (Fig. 4A). Diabetes significantly impaired LTP formation (Fig. 4C; *P* < 0.0001 vs. Control-Vehicle), which was restored by bath application with AdipoRon (10 min; 0.5 μM) (Fig. 4C; *P* < 0.0001 vs. STZ). Two-way ANOVA revealed significant effects of AdipoRon and diabetic on LTP formation (Fig. 4B and C: Effect of STZ-diabetic: *F*_*1,40*_ = 26.25, *P* < 0.0001; effect of AdipoRon: *F*_*1,40*_ = 23.08, *P* < 0.0001; effect of interaction: *F*_*1,40*_ = 12.69, *P* = 0.001). PGC-1α is a master regulator of mitochondrial biogenesis (66, 67) which is necessary for LTP formation (68). As expected, pharmacological inhibition of PGC-1α by SR-18292 (Fig. 4D) diminished the promoting effect AdipoRon on LTP formation in diabetic mice (Fig. 4E & 4F; *P* = 0.514 vs. STZ-AdipoRon**)**. Bath application with NMDA receptor subunit blockers, including NVP-AAM077 (GluN2A inhibitor) or Ro 25-6981 (GluN2B inhibitor) (Fig. 4G), showed similar effects (Fig. 4H and I; GluN2A: *P* < 0.0188 vs. STZ-AdipoRon; GluN2B: *P* < 0.011 vs. STZ-AdipoRon). One-way ANOVA revealed significant effects of pharmacological inhibitions of NMDAR subunits on LTP formation (*F*_*2,27*_ = 6.133, *P* = 0.0064). Altogether, the findings demonstrated the involvement of PGC-1α and NMDA receptor subunits GluN2A and GluN2B in the action of AdipoRon on restoring synaptic plasticity in diabetic mice.

### AdipoRon Treatment Promoted BDNF Levels and Activated AMPK/PGC-1α Signalling in the Hippocampal DG

**Fig. 5:**
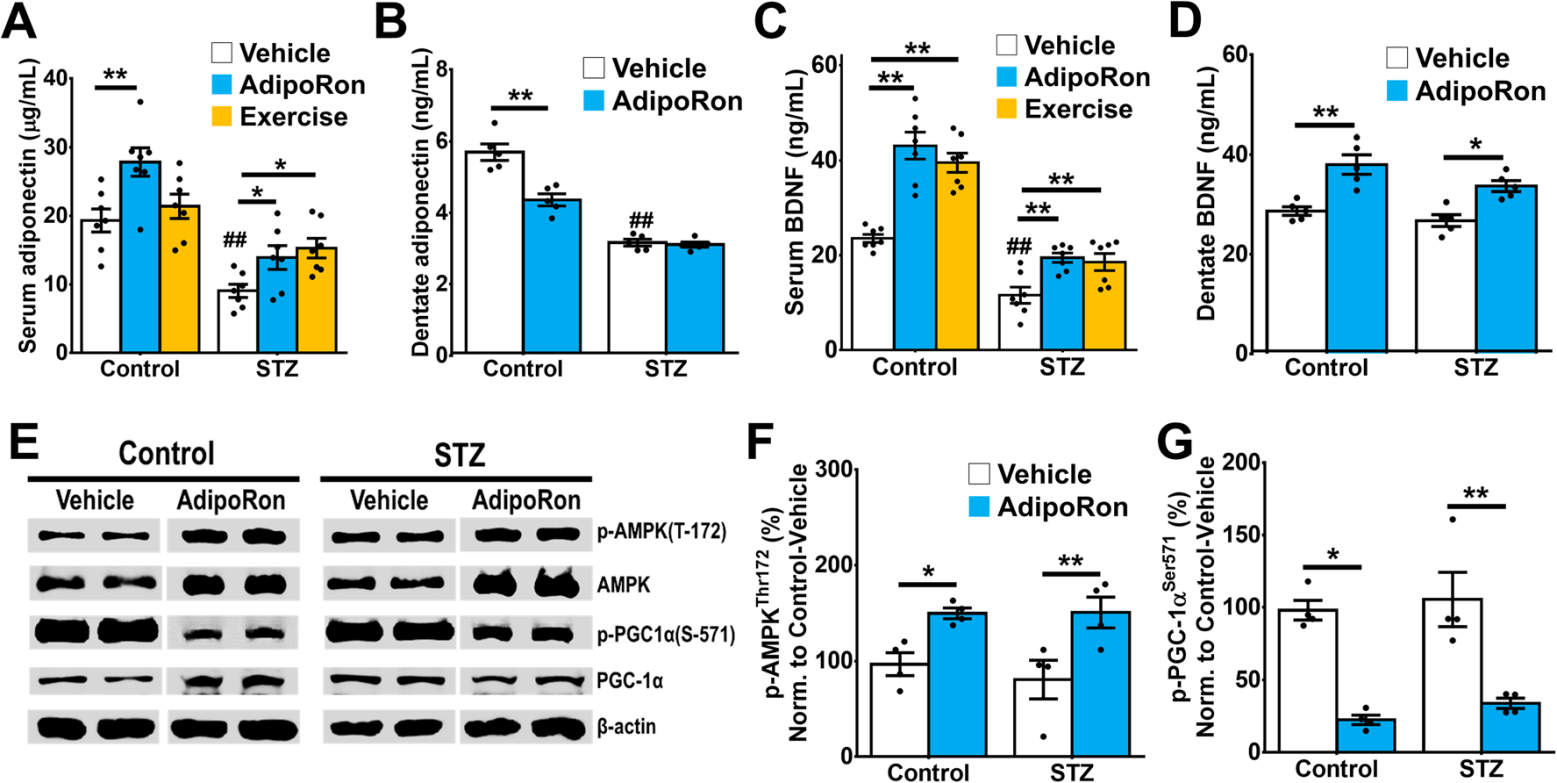
Effects of AdipoRon treatment on expression levels of adiponectin, BDNF, and activation of AMPK and PGC-1α signalling. **A** STZ-diabetes reduced serum adiponectin levels (Tukey’s post hoc test: ^##^*P* < 0.005 vs. Control-Vehicle). AdipoRon treatment restored serum adiponectin levels in diabetic mice, as voluntary running did (Tukey’s post hoc test: *P < 0.05 vs. STZ-Vehicle). AdipoRon treatment increased serum adiponectin levels in control mice (Tukey’s post hoc test: **P < 0.005 vs. Control-Vehicle) while exercise could not (Tukey’s post hoc test: *P >* 0.05 vs. Control-Vehicle). *n* = 7 per group. Previously reported Control-Vehicle and Control-AdipoRon data were used to support this study (44). **B** Diabetes significantly reduced adiponectin level in DG (Tukey’s post hoc test: ^##^*P* < 0.005 vs. Control-Vehicle), whereas AdipoRon treatment reduced adiponectin levels in control (Tukey’s post hoc test: **P < 0.005 vs. Control-Vehicle). AdipoRon treatment did not restore adiponectin levels in DG from diabetic mice. *n* = 5 replicates. **C** Diabetes reduced serum BDNF levels (Tukey’s post hoc test: ^##^*P* < 0.005 vs. Control-Vehicle). AdipoRon and exercise treatment increased serum BDNF levels in control animals (Tukey’s post hoc test: **P < 0.005 vs. Control-Vehicle), while both treatments restored serum BNDF levels in diabetic mice (Tukey’s post hoc test: **P < 0.005 vs. STZ-Vehicle). *n* = 7 per group. Previously reported Control-Vehicle and Control-AdipoRon data were used to support this study (44). **D** Diabetes did not alter BDNF level in DG, whereas AdipoRon treatment increased BDNF levels in control (Tukey’s post hoc test: **P < 0.005 vs. Control-Vehicle) and diabetic mice (Tukey’s post hoc test: *P < 0.05 vs. STZ-Vehicle). *n* = 5 replicates. **E** Representative images of western blot analysis. (*n* = 4 technical replicates from 7 pairs of DG pooled-sample/group). **F** AdipoRon treatment also increased p-AMPKα^Thr172^ expression in the hippocampal DG (LSD post hoc test: *P < 0.05 vs. Control-Vehicle). **G** AdipoRon treatment significantly suppressed the expressions of p-PGC-1α^Ser571^, a phosphorylated inhibitory form of PGC-1α, in the DG of control (LSD post hoc test: *P < 0.05 vs. Control-Vehicle) and diabetic mice (LSD post hoc test: ** *P* < 0.05 vs. STZ-Vehicle)

Voluntary running exercise elevates adiponectin levels in the whole hippocampus but not in the serum of healthy mice (69). Our results showed that AdipoRon treatment restored serum adiponectin levels in diabetic mice (Fig. 5A; STZ-AdipoRon: *P* = 0.0434 vs. STZ-Vehicle) resembling the effect of voluntary wheel running (Fig. 5A; STZ-Exercise: *P* = 0.011 vs. STZ-Vehicle). However, AdipoRon treatment did not restore the decrease in adiponectin level in the DG of diabetic mice (Fig. 5B; *P* = 0.994 vs. STZ-Vehicle) and reduced adiponectin levels in the control mice (Fig. 5B; *P* < 0.0001 vs. Control-Vehicle).

BDNF is a critical neurotrophin required for physical exercise to induce hippocampal neurogenesis. We examined whether AdipoRon enhances BDNF levels and AMPK/PGC-1α signalling that is also be activated by physical exercise (34, 39, 70). AdipoRon and exercise treatment elevated BDNF levels in the control mice (Fig. 5C; Control-AdipoRon: *P* < .0001 and Control-Exercise: *P* < 0.0001 vs. Control-Vehicle). Diabetic condition reduced serum BDNF levels (Fig. 5C; STZ-Vehicle *P* < 0.0001 vs. Control-Vehicle). AdipoRon and voluntary running restored serum BDNF levels in diabetic mice (Fig. 5C; STZ-AdipoRon: *P* = 0.0039 and STZ-Exercise: *P* = 0.0098 vs. STZ-Vehicle). Apart from the elevated serum BDNF levels following AdipoRon treatment, BDNF levels in the hippocampal DG were increased, though diabetes did not alter BDNF levels in the dentate region (Fig. 5D; *P* = 0.743 vs. Control-Vehicle). Conversely AdipoRon treatment promoted BDNF levels in the DG of diabetic (Fig. 5D; *P* = 0.0039 vs. STZ-Vehicle) and control mice (Fig. 5D; *P* < 0.0001 vs. Control-Vehicle). These findings implicated that increased BDNF levels could be linked to the promoting effect of AdipoRon on hippocampal neuroplasticity.

We have previously shown that adiponectin mediates physical exercise-induced adult hippocampal neurogenesis through AdipoR1/AMP-activated protein kinase (AMPK). Besides, AMPK and PPAR-γ coactivator 1-α (PGC-1α) are potential upstream transducers of BDNF; therefore, we further tested whether AdipoRon elicited its effect via the AMPK/PGC-1α pathway (Fig. 5E). Western blot analysis showed that AdipoRon treatment enhanced the phosphorylation of AMPK^Thr172^ (Fig. 5F) in both diabetic and control mice. AdipoRon treatment also activated PGC-1 α in diabetic and control mice, as indicated by reducing the inhibitory form of PGC-1α^Ser571^ (Fig. 5G). In sum, the results suggested that AdipoRon treatment significantly increased AMPK and PGC-1α activity and BDNF levels in the DG.

## Discussion

This study demonstrates that AdipoRon treatment elicits pro-cognitive and neurotrophic effects indicating its potential in acting as an exercise mimetic. Here we demonstrated that AdipoRon treatment rescued spatial memory deficits in diabetic mice in a manner that was reminiscent of the pro-cognitive effects observed with voluntary running exercise (see also Appendix Tables S1 and S2). AdipoRon also rescued adult hippocampal neurogenesis, increased dendritic arborisation and spine density, enhanced hippocampal LTP, and increased serum and hippocampal DG levels of BDNF in diabetic mice.

The antidiabetic effects of AdipoRon were first demonstrated in high-fat diet-fed mice and leptin-deficient (*db*/*db*) mice (42). In this study, we used a diabetic mouse model presenting more pronounced cognitive impairments to examine the therapeutic effects of AdipoRon for cognition. We have previously reported that streptozotocin-induced diabetes impairs adult hippocampal neurogenesis (30). Our results support a prior study that a single injection of streptozotocin (65 mg/kg i.v.) can induce hippocampal atrophy, spine loss, and hippocampal-dependent spatial learning and memory impairment (71).

Physical exercise facilitates learning and memory formation, enhances adult hippocampal neurogenesis, increases dendritic arborisation, and improves hippocampal synaptic plasticity (72–76). In addition to promoting adult hippocampal neurogenesis (33) and learning and memory performance (32), adiponectin can mediate the effects of physical exercise on reducing depression and promoting hippocampal cell proliferation (30, 34). In the current study, we showed that the adiponectin receptor agonist, AdipoRon, could similarly restore hippocampal cell proliferation, BDNF levels, and memory performance in diabetic mice (34, 62). Furthermore, AdipoRon treatment restored dendritic atrophy and spine density in DG granule cells of the diabetic mice. Adiponectin has shown to be indispensable for dendritic and spine formation in the DG granule neurons (33). Our results showed that AdipoRon treatment promoted dendritic complexities and spine densities in the dentate granule neurons associated with the increased serum and dentate BDNF levels, whereas diabetic mice showed a significant reduction in serum and dentate adiponectin levels. AD mice show decreased adiponectin levels and spine density in the cortex and hippocampus, which could be restored by AdipoRon treatment (31). Our results collectively suggest that AdipoRon treatment resembles the pro-cognitive and neurotrophic effects of voluntary running and could be an effective treatment for restoring structural plasticity and improving learning and memory associated with diabetes.

Both adiponectin and AdipoRon can cross the blood-brain barrier (31, 34). To examine the direct effect of AdipoRon, we showed that AdipoRon, like adiponectin, can elicit direct proliferative effects in the N2a cultured cell, where adiponectin receptors are expressed (34). The current results have demonstrated the direct effect of AdipoRon on restoring LTP formation in the dentate region in diabetic mice. It is known that adiponectin modulates LTP formation in the hippocampal CA1 (8) and CA3 (32) regions, whereas AdipoRon can restore LTP deficits induced by adiponectin deficiency (32). Our findings indicated the significant effect of AdipoRon on restoring synaptic deficits in the hippocampus, a brain region with AdipoR1 and AdipoR2 being expressed (32). NMDA receptor (NMDAR) subunits, such as GluN2A and GluN2B, mediate the LTP induction in the hippocampus (77, 78), while adiponectin deficiency reduces the expressions of the α-amino-hydroxy-methyl-isoxazolepropionic acid receptor (AMPAR) and NMDAR subunits in the hippocampus (79). The expressions of AMPAR and NMDAR subunits and adiponectin receptors are increased in post-recording 5×FAD hippocampal slices (80). The present study also addressed the critical roles of GluN2A/B in AdipoRon-induced LTP formation in hippocampal slices from diabetic mice, suggesting the involvement of NMDA receptors in AdipoRon-induced synaptic plasticity in the hippocampus.

AdipoRon might exert indirect action on promoting hippocampal plasticity because serum adiponectin levels in diabetes were increased by AdipoRon treatment. Adiponectin levels in the DG were markedly reduced in diabetic mice, and AdipoRon treatment failed to restore the decrease. Besides, AdipoRon treatment reduced adiponectin levels in the DG in physiological condition. A feedback mechanism may be involved in downregulating the adiponectin levels in the brain in response to the AdipoRon action on activating central adiponectin signalling. In contrast, excessive adiponectin in the brain or hyperactivation of the adiponectin signalling pathway may impair neural plasticity. Another possibility is that AdipoRon may increase the expression levels of adiponectin receptors in the hippocampal DG. Therefore, the reduction in adiponectin levels serves as a negative feedback regulation to avoid over-activation of adiponectin signalling. Lastly, adiponectin receptors are expressed on endothelial cells of the blood-brain barrier (BBB) (81), and adiponectin signalling maintains the BBB integrity (82). AdipoRon administration and elevated circulating adiponectin may alter the BBB permeability, limiting the entry of adiponectin to the brain from the circulation.

**Fig. 6:**
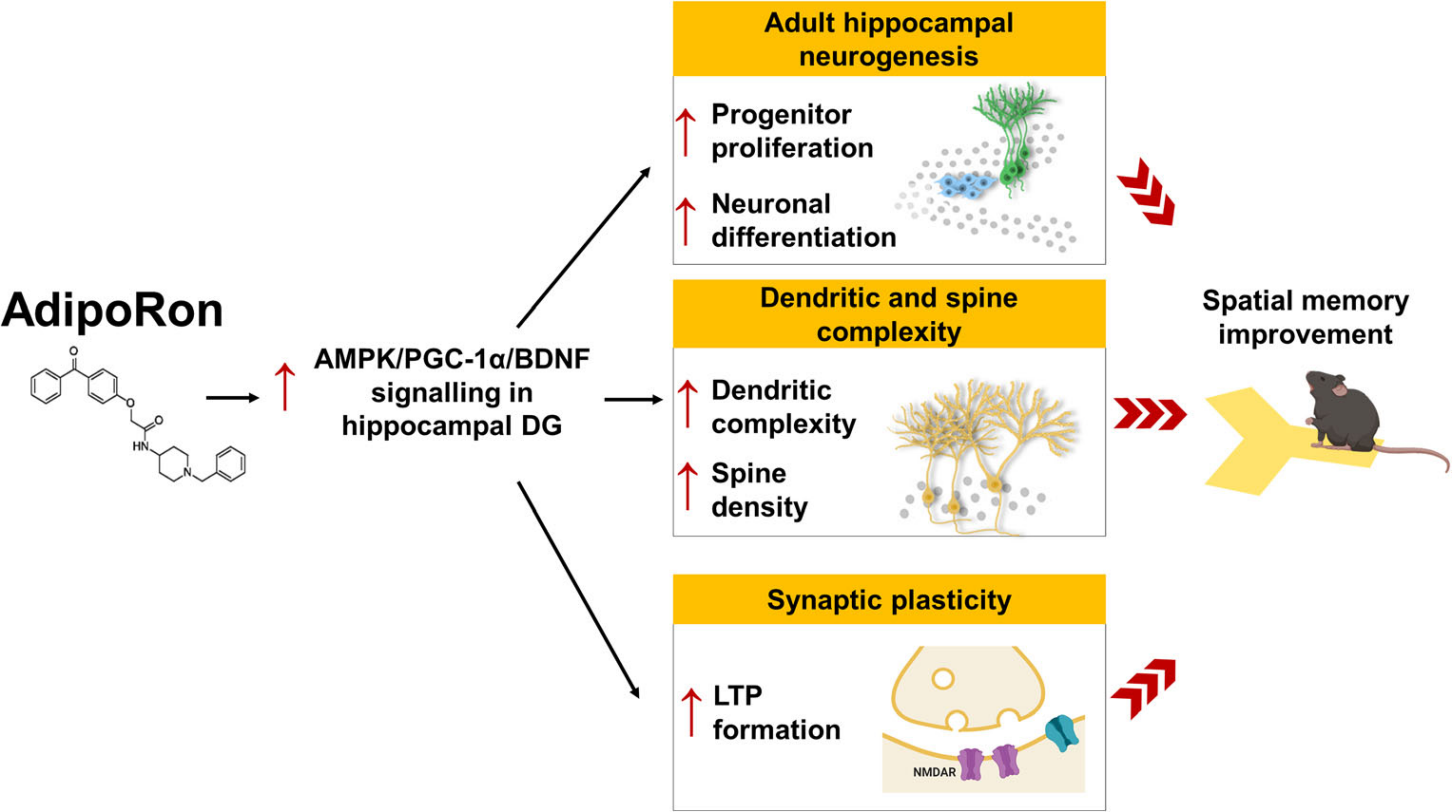
Summary of the effects of AdipoRon on promoting hippocampal neuroplasticity. Two weeks of AdipoRon treatment significantly promoted cell proliferation and neuronal differentiation in the dentate region. AdipoRon restored diabetes-induced impairment in adult hippocampal neurogenesis, dendritic complexity, and spine density, as well as NMDA receptor-dependent synaptic plasticity, through activating AMPK/PGC-1α/BDNF signalling in the hippocampus. Graphical images are created in BioRender©

Elevated BDNF levels promote adult neurogenesis (83) and synaptic plasticity (84), which in turn facilitate learning and memory formation (85). Our findings demonstrated that enhancement in structural and synaptic plasticity is associated with elevated dentate and serum BDNF levels following AdipoRon treatment. The central BDNF levels are known to be positively correlated with peripheral BDNF levels (86). Physical exercise promotes hippocampal BDNF levels through PGC-1α signalling (39). Over-expression of PGC-1α restores BDNF levels in Aβ-treated N2a cells (87). We have previously shown that adiponectin is required for exercise to induce AMPK activation in the hippocampus (34); however, administration of AdipoRon activated AMPK/PGC-1α signalling without increasing adiponectin levels in diabetic mice, suggesting a direct action of AdipoRon on activating adiponectin receptors and thus its downstream signalling cascade. The beneficial effects of AdipoRon on restoring hippocampal neuroplasticity could be linked to the activation of AMPK/PGC-1α signalling pathway (Fig. 6). Our study reported that AdipoRon treatment enhances PGC-1α activity in the DG. The in vitro proliferative assay has suggested the involvement of PGC-1α/PPAR-γ in the AdipoRon-induced cell proliferation and the involvement of PGC-1α in the AdipoRon-induced LTP formation in diabetic mice. PGC-1α mediates the AMPK-induced neuronal growth and maturation (88). PGC-1α is also essential for spine formation (40) and is a master regulator of mitochondrial biogenesis (66). Mitochondrial Ca^2+^ uptake is involved in LTP formation in the hippocampus (68). It is possible that AdipoRon could activate PGC-1α activity to promote mitochondrial biogenesis, which in turn promotes Ca^2+^ influx; and facilitates LTP formation.

Furthermore, AMPK is a master regulator of metabolism (89) and is necessary for maintaining neural stem cell proliferation (90). Voluntary running promotes hippocampal AMPK activation (40), which is absent in adiponectin knockout mice (34). Conversely, adiponectin deficiency reduces hippocampal AMPK activation (32). Activating AMPK activity leads to increased BDNF levels in a dose-dependent manner (91). We reported that AdipoRon treatment increased the serum adiponectin levels, but not in the dentate gyrus in diabetic mice, suggesting the activation of AMPK in the dentate region could be a direct effect of AdipoRon. Taken together, our results have suggested the involvement of AMPK/PGC-1α/BDNF signalling in the action of AdipoRon on rescuing hippocampal atrophy in diabetic mice.

## Conclusion

Our results demonstrated that chronic treatment with AdipoRon mimicked the beneficial effects of physical exercise on promoting hippocampal-dependent learning and memory function and restoring adult neurogenesis in diabetic condition. In addition, AdipoRon treatment promoted dendritic complexity and synaptic plasticity as well as serum and dentate BDNF levels in the hippocampus of diabetic mice. The beneficial effects could be dependent on downstream AMPK/PGC-1α signalling. An adiponectin-based therapeutic which can enhance hippocampal BDNF levels could serve as a pharmacological intervention for restoring diabetes-associated learning and memory impairment.

## Supplementary Information

Fig. S1 (PNG 1321 kb)

High Resolution (TIF 392 kb)

Fig. S2 (PNG 41390 kb)

High Resolution (TIF 9487 kb)

Fig. S3 (PNG 13384 kb)

High Resolution (TIF 3801 kb)

Fig. S4 (PNG 3183 kb)

High Resolution (TIF 1140 kb)

Appendix Table S1 (PNG 1074 kb)

High Resolution (TIFF 345 kb)

Appendix Table S2 (PNG 1088 kb)

High Resolution (TIFF 357 kb)

## Data Availability

Data are available upon request.
