# Supplementary figures and images for: Chronic AdipoRon Treatment Mimics the Effects of Physical Exercise on Restoring Hippocampal Neuroplasticity in Diabetic Mice
Source: Mol Neurobiol. 2021 Jun 23;58(9):4666–81. doi: 10.1007/s12035-021-02441-7 (PMC8487422; doi:10.1007/s12035-021-02441-7)

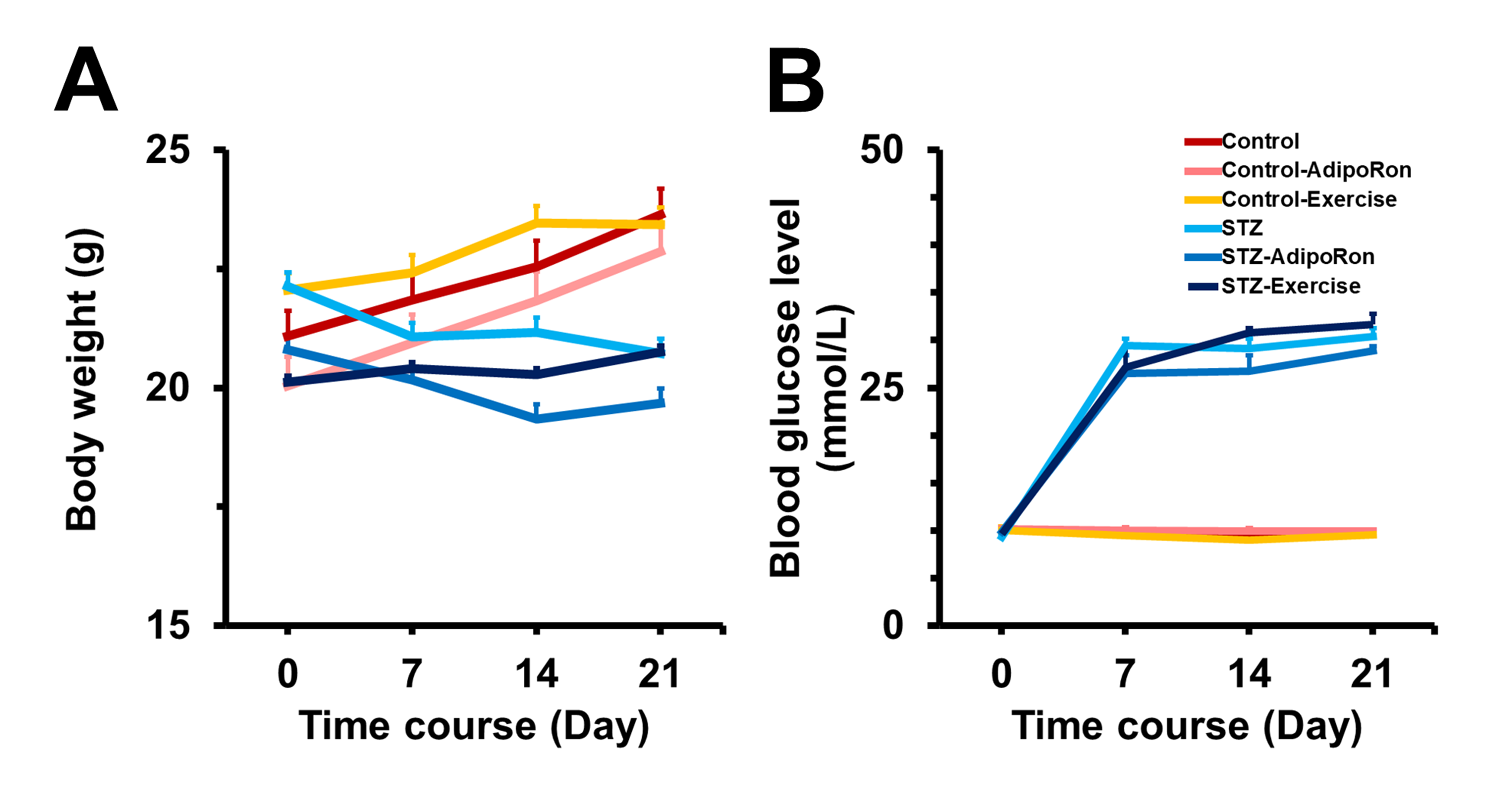

Supplement: Supplementary file 1 — (PNG 1321 kb) [file 12035_2021_2441_Fig7_ESM.png]

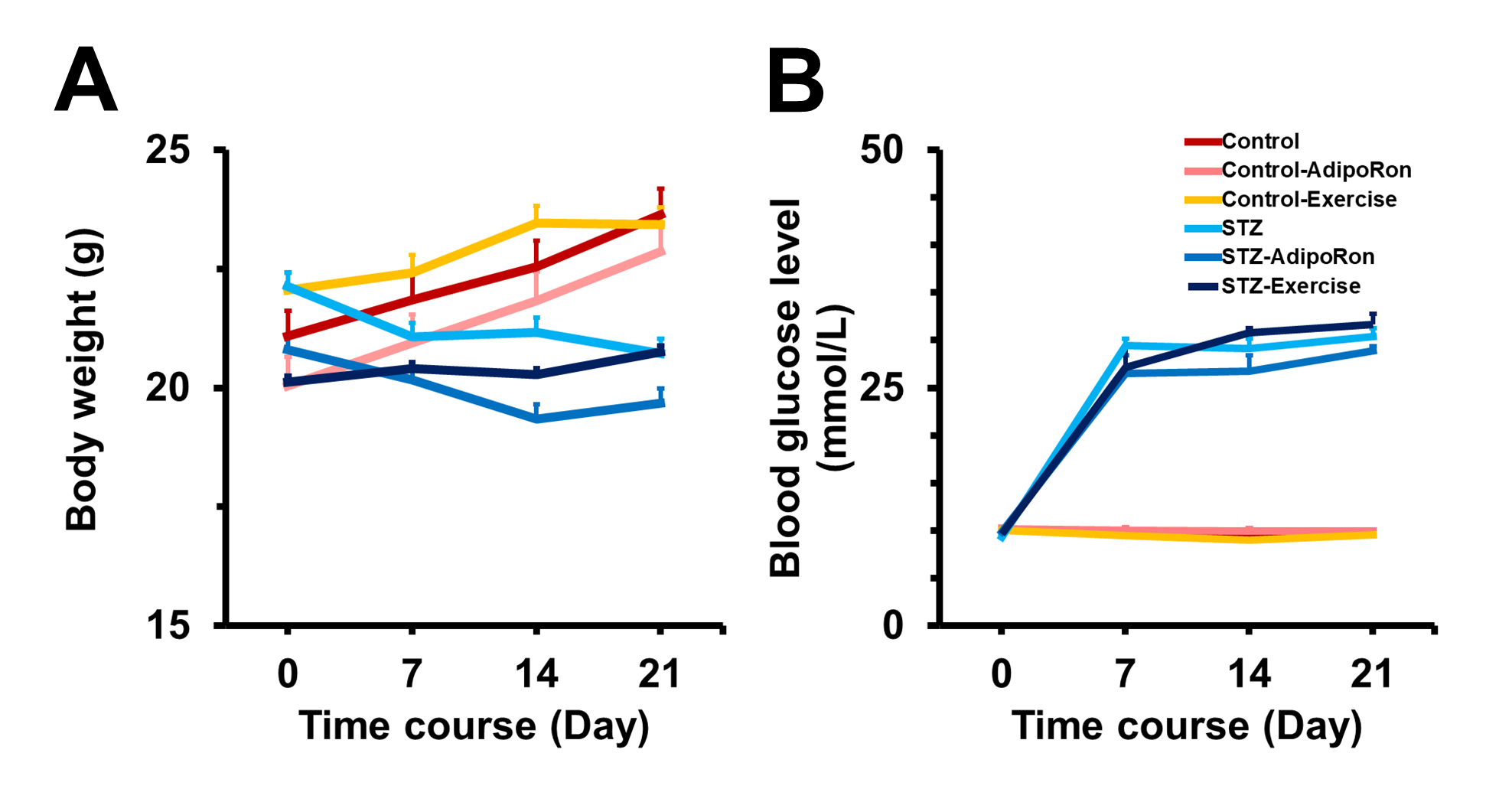

Supplement: Supplementary file 2 — High Resolution (TIF 392 kb) [file 12035_2021_2441_MOESM1_ESM.tif]

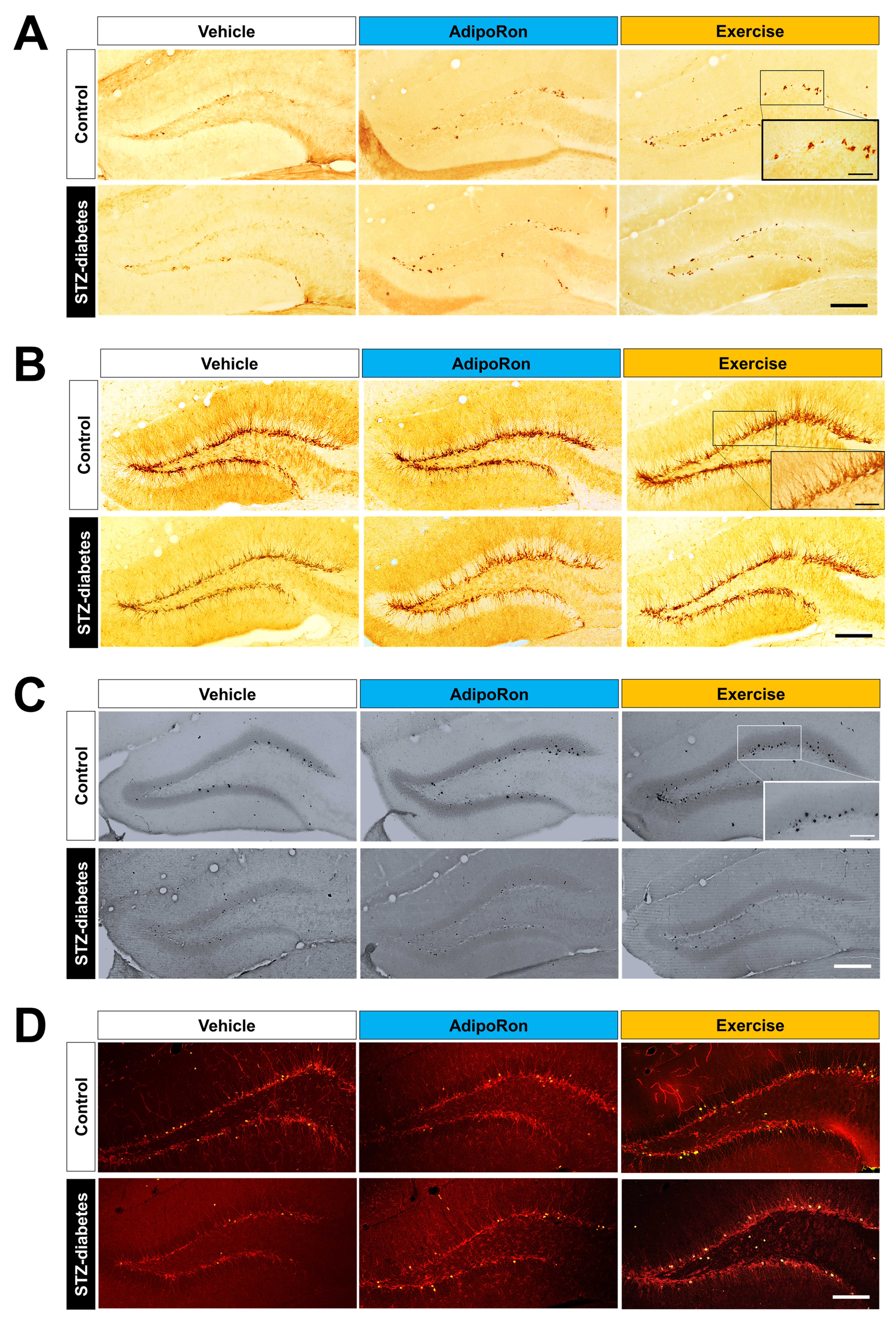

Supplement: Supplementary file 3 — (PNG 41390 kb) [file 12035_2021_2441_Fig8_ESM.png]

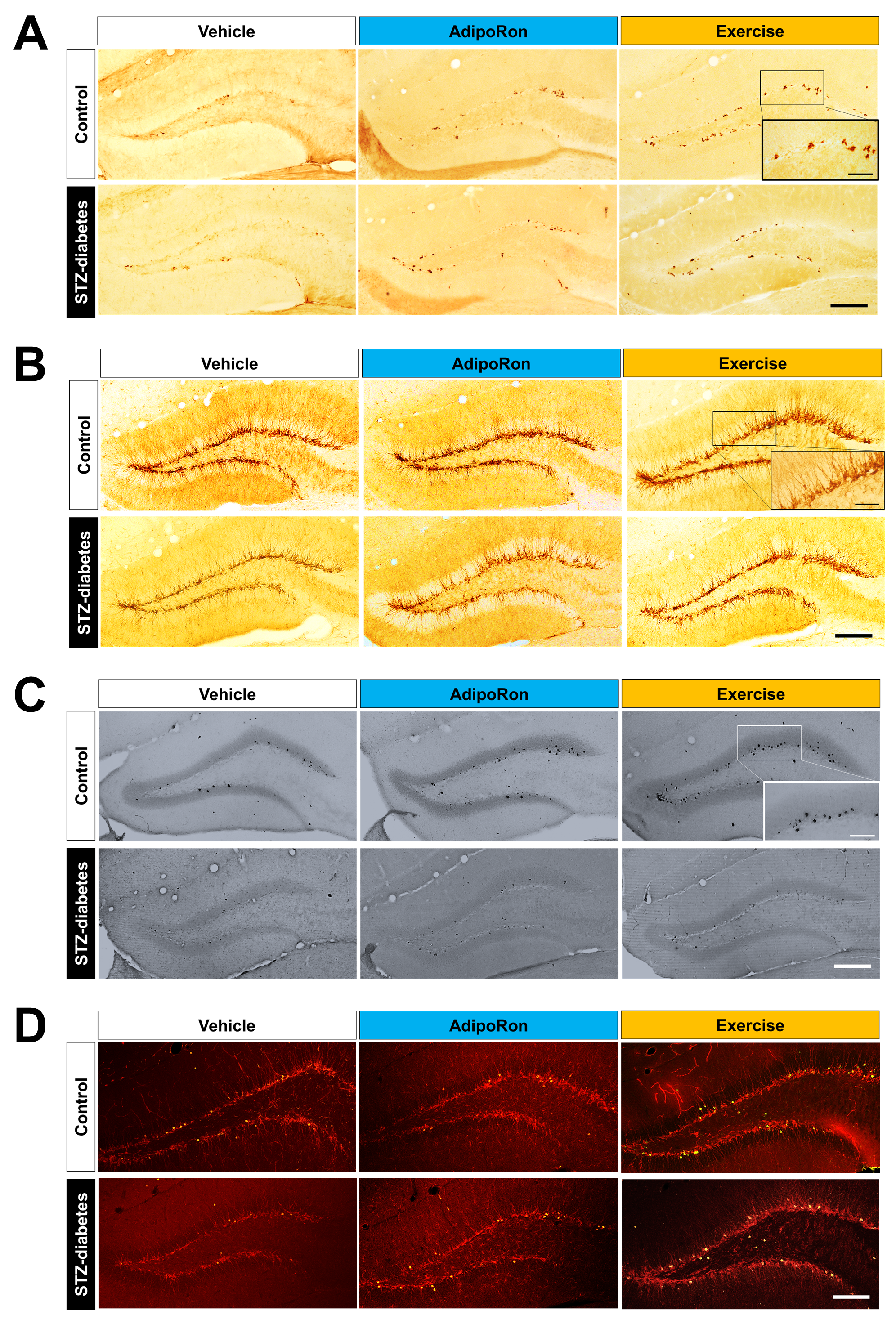

Supplement: Supplementary file 4 — High Resolution (TIF 9487 kb) [file 12035_2021_2441_MOESM2_ESM.tif]

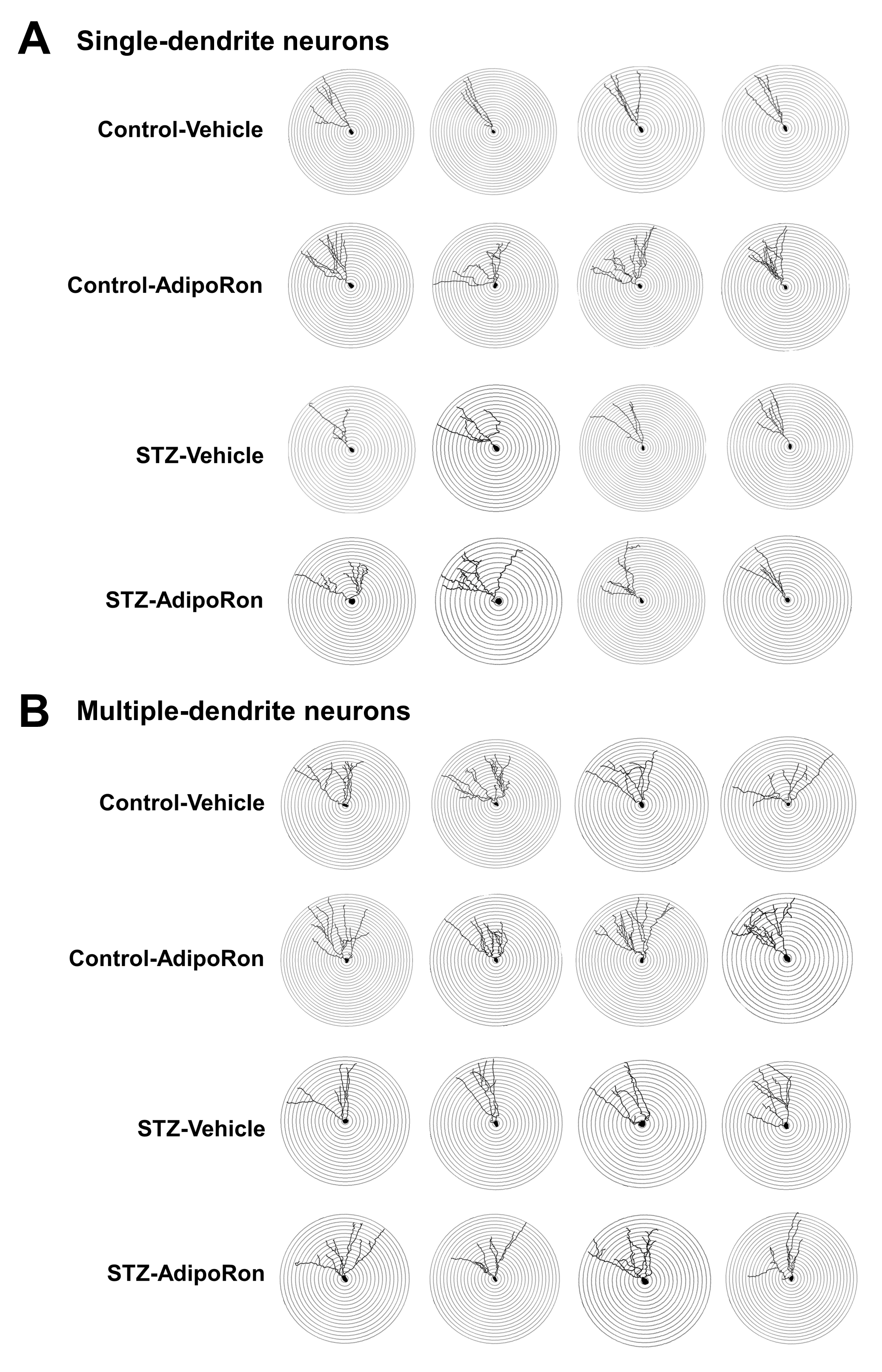

Supplement: Supplementary file 6 — High Resolution (TIF 3801 kb) [file 12035_2021_2441_MOESM3_ESM.tif]

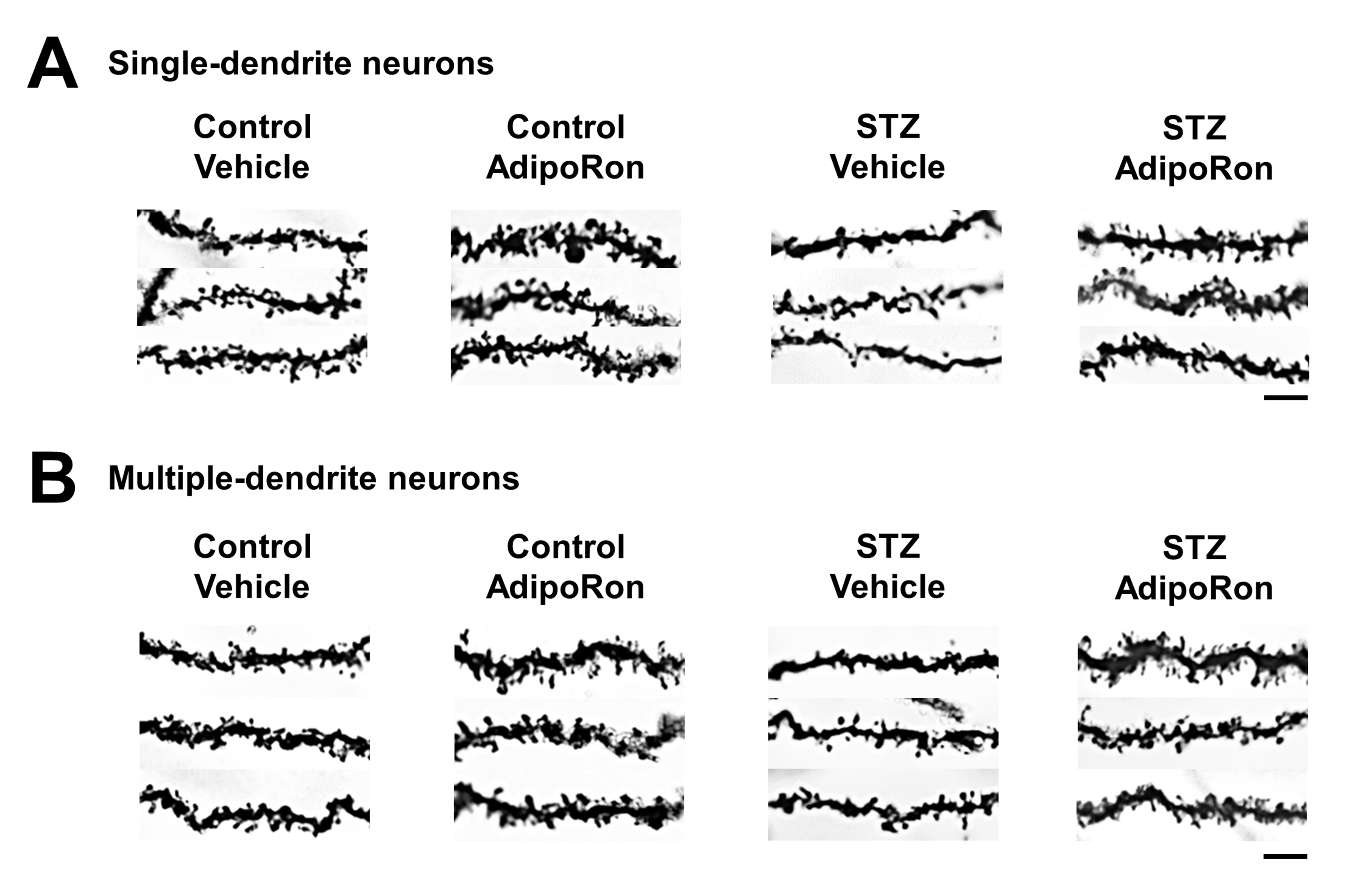

Supplement: Supplementary file 7 — (PNG 3183 kb) [file 12035_2021_2441_Fig10_ESM.png]

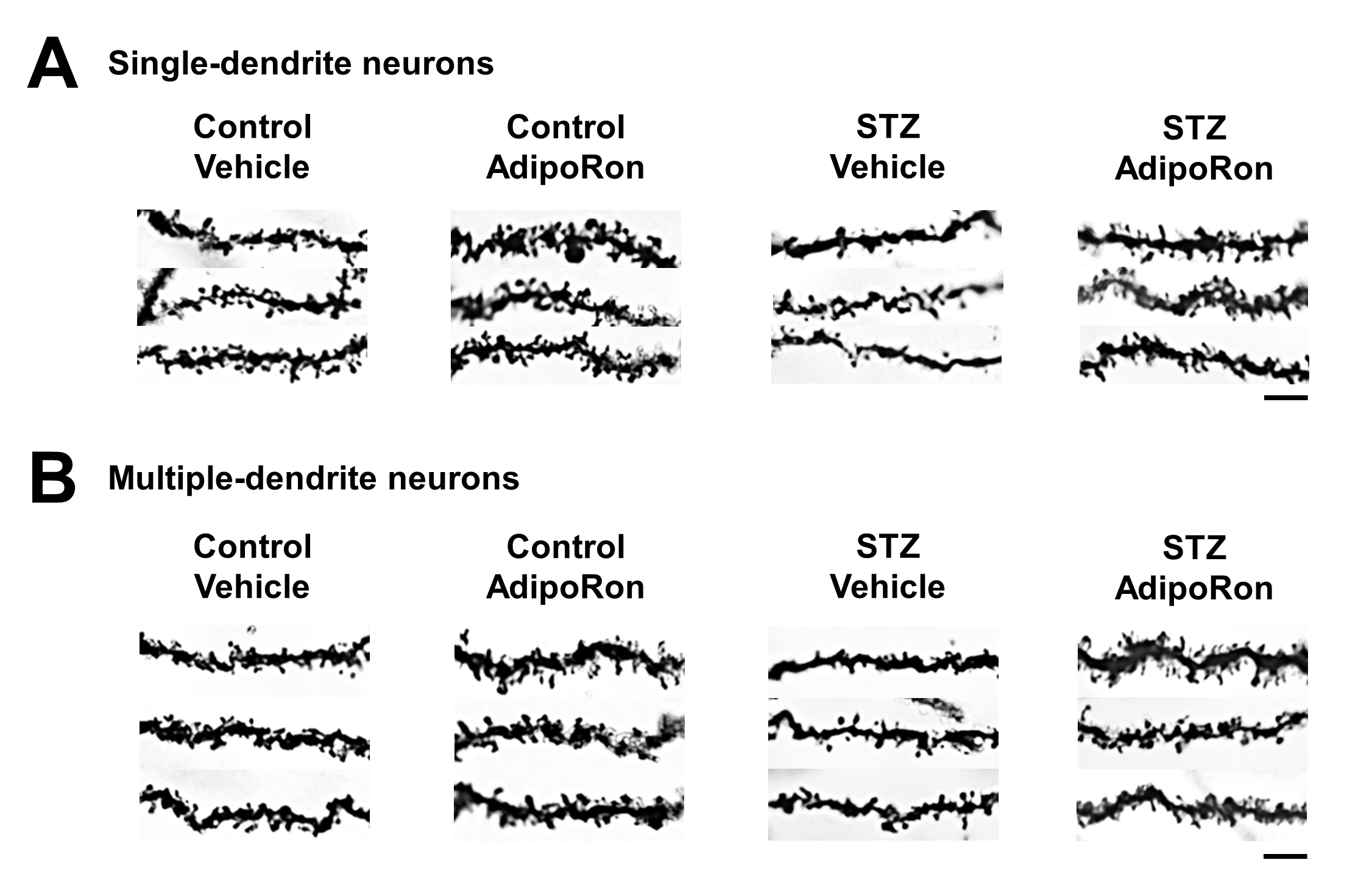

Supplement: Supplementary file 8 — High Resolution (TIF 1140 kb) [file 12035_2021_2441_MOESM4_ESM.tif]

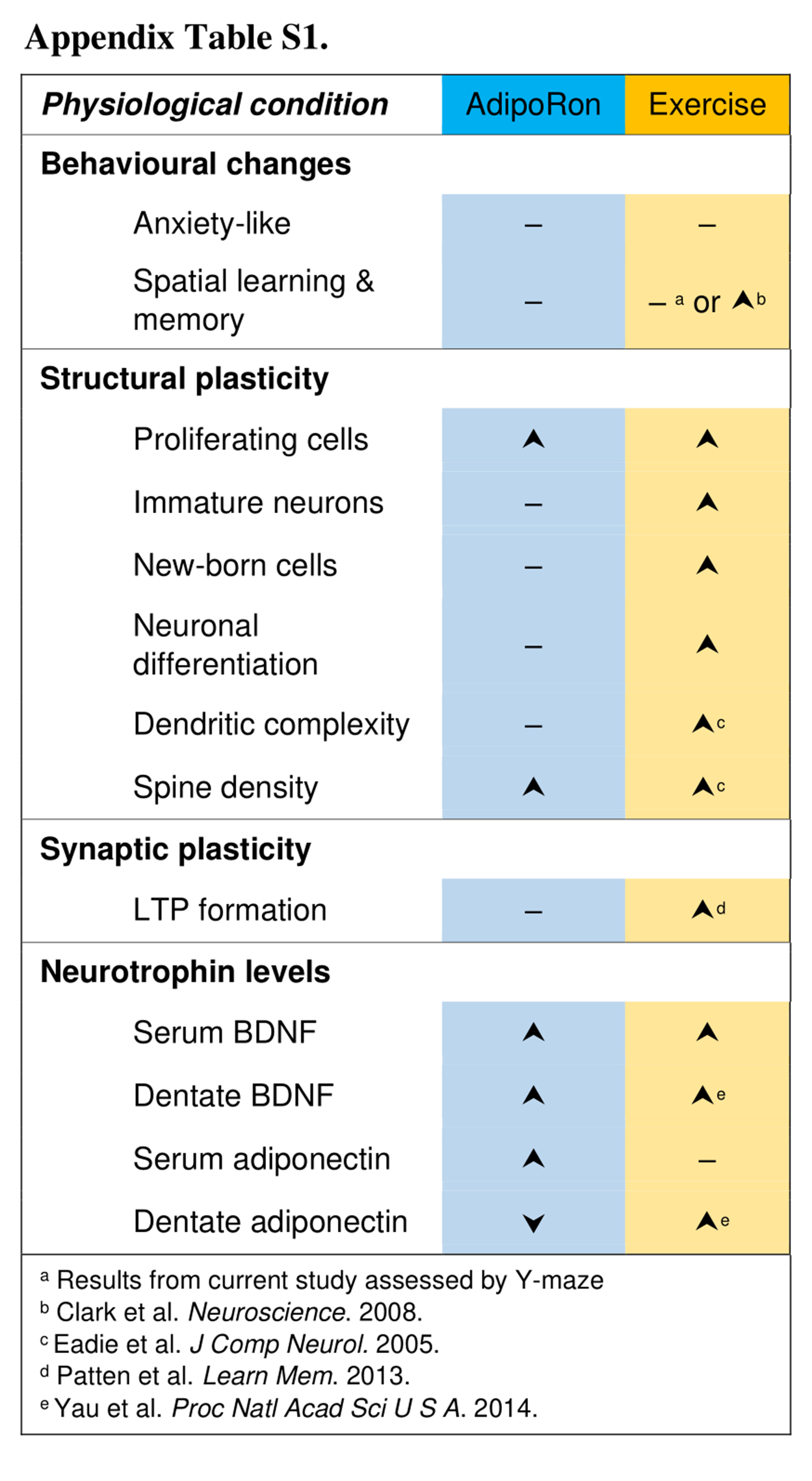

Supplement: Supplementary file 9 — (PNG 1074 kb) [file 12035_2021_2441_Fig11_ESM.png]

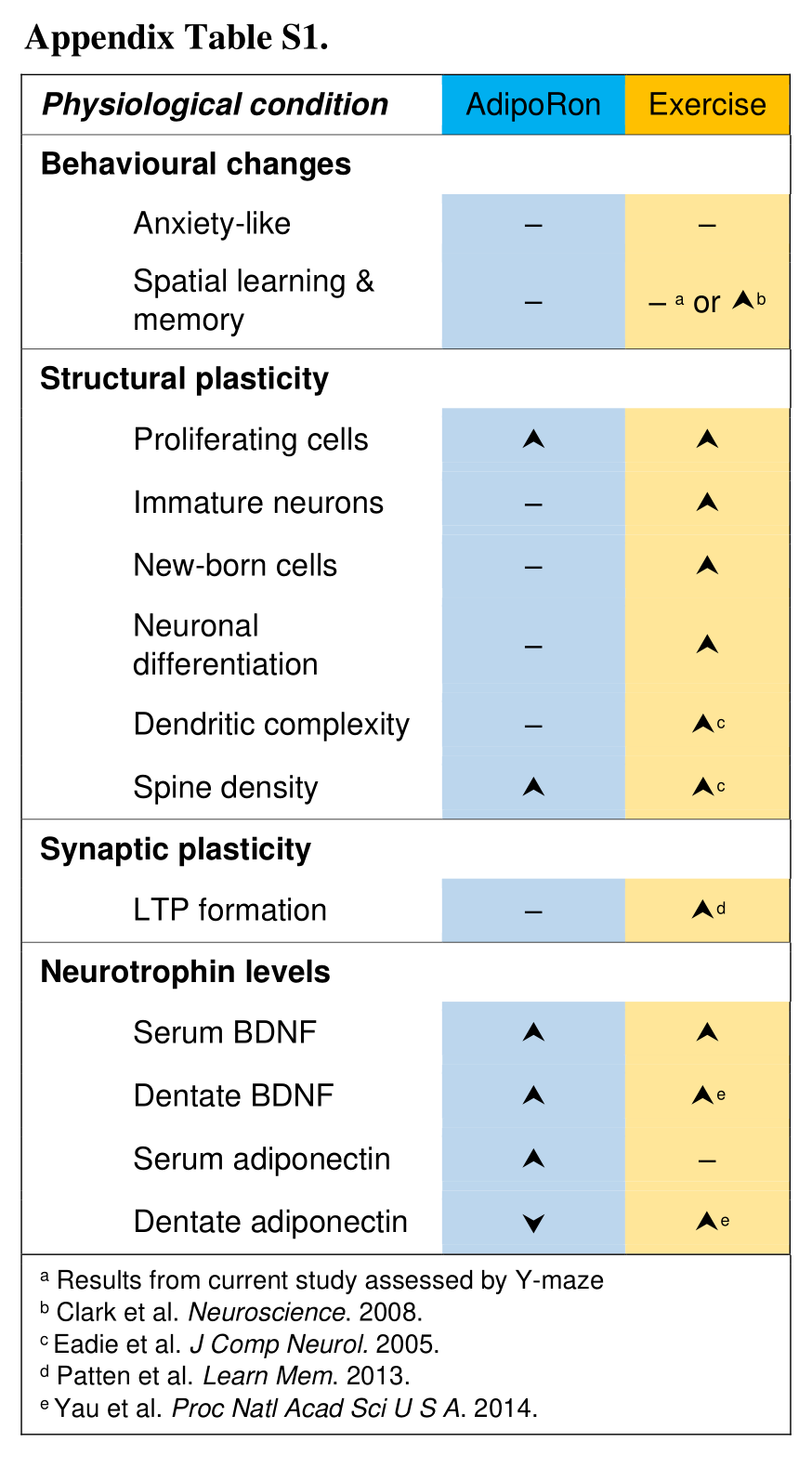

Supplement: Supplementary file 10 — High Resolution (TIFF 345 kb) [file 12035_2021_2441_MOESM5_ESM.tiff]

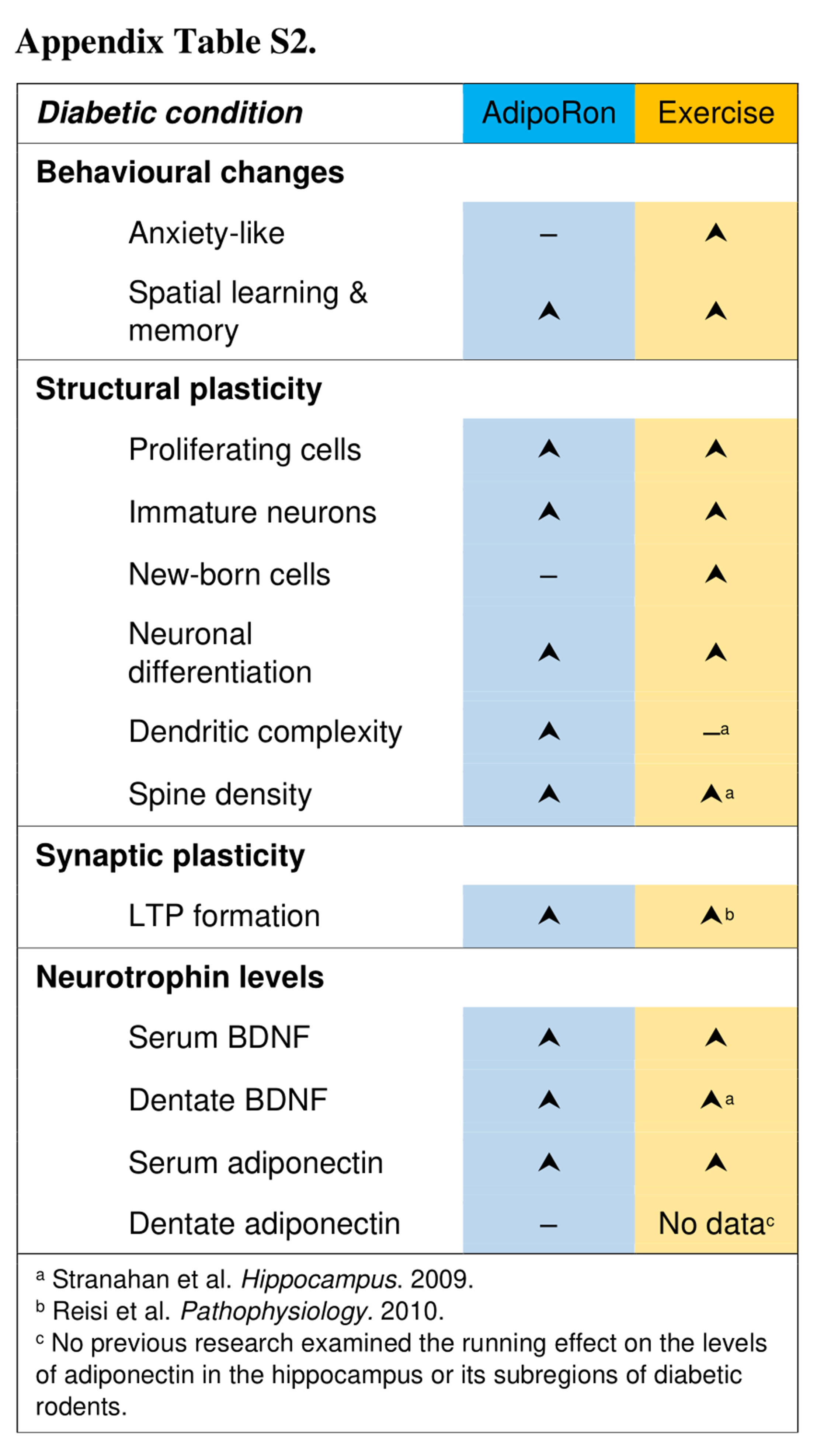

Supplement: Supplementary file 11 — (PNG 1088 kb) [file 12035_2021_2441_Fig12_ESM.png]

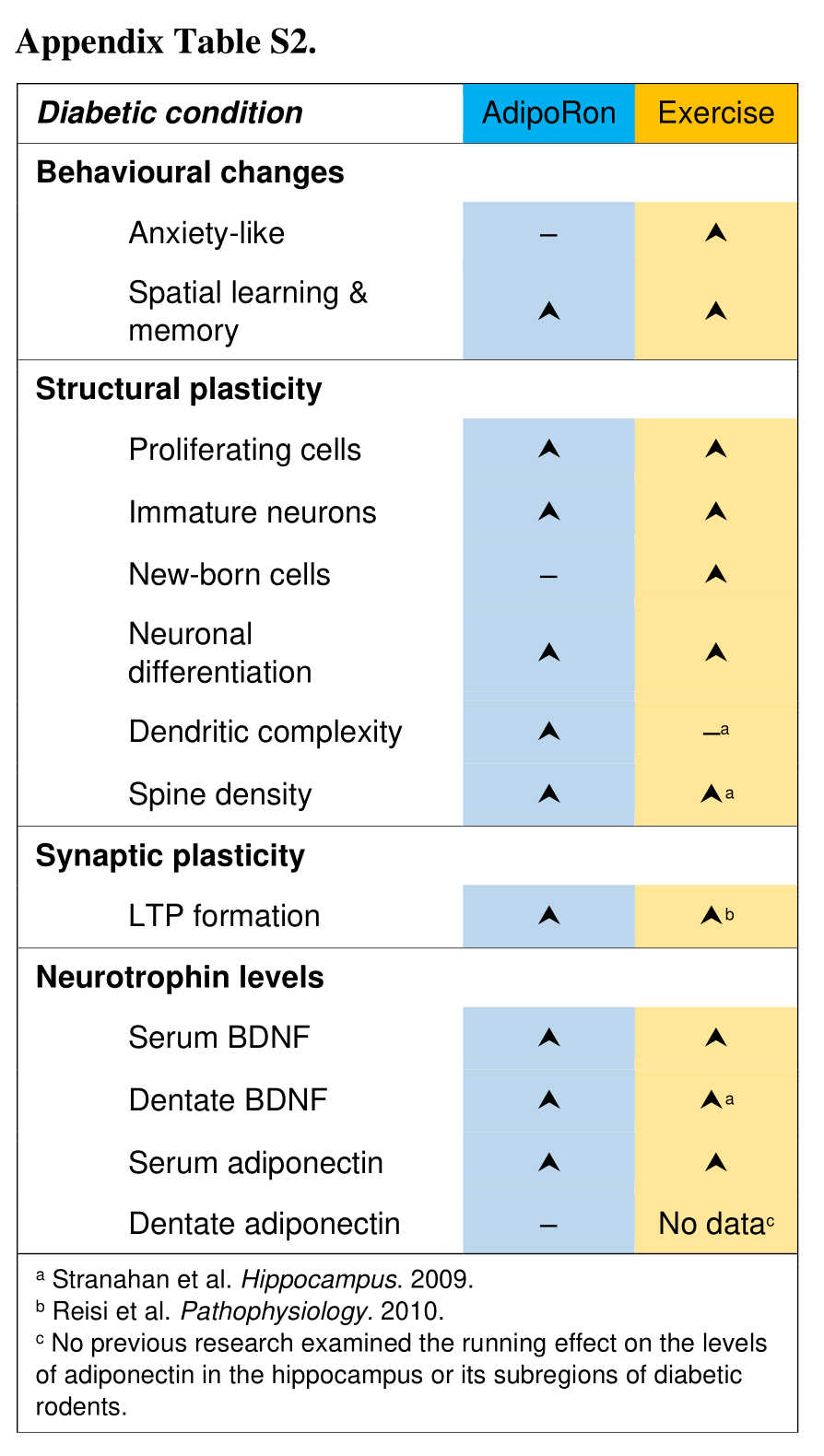

Supplement: Supplementary file 12 — High Resolution (TIFF 357 kb) [file 12035_2021_2441_MOESM6_ESM.tiff]
